# The Impact of Patient Infection Rate on Emergency Department Patient Flow: Hybrid Simulation Study in a Norwegian Case

**DOI:** 10.3390/healthcare11131904

**Published:** 2023-06-30

**Authors:** Gaute Terning, Idriss El-Thalji, Eric Christian Brun

**Affiliations:** 1 Department of Safety, Economics, and Planning, University of Stavanger, 4036 Stavanger, Norway; 2 Department of Mechanical and Structural Engineering and Materials Science, University of Stavanger, 4036 Stavanger, Norway

**Keywords:** healthcare, emergency department, patient flow, patient infection rate, COVID-19 pandemic, agent-based hybrid model, multi-agent hybrid model, pandemic decision support

## Abstract

The COVID-19 pandemic put emergency departments all over the world under severe and unprecedented distress. Previous methods of evaluating patient flow impact, such as in-situ simulation, tabletop studies, etc., in a rapidly evolving pandemic are prohibitively impractical, time-consuming, costly, and inflexible. For instance, it is challenging to study the patient flow in the emergency department under different infection rates and get insights using in-situ simulation and tabletop studies. Despite circumventing many of these challenges, the simulation modeling approach and hybrid agent-based modeling stand underutilized. This study investigates the impact of increased patient infection rate on the emergency department patient flow by using a developed hybrid agent-based simulation model. This study reports findings on the patient infection rate in different emergency department patient flow configurations. This study’s results quantify and demonstrate that an increase in patient infection rate will lead to an incremental deterioration of the patient flow metrics average length of stay and crowding within the emergency department, especially if the waiting functions are introduced. Along with other findings, it is concluded that waiting functions, including the waiting zone, make the single average length of stay an ineffective measure as it creates a multinomial distribution of several tendencies.

## 1. Introduction

The COVID-19 pandemic has placed a significant burden on the healthcare system since early 2020 and was pronounced by the world health organization (WHO) to constitute a global public health emergency of international concern [1]. Emergency departments had to amend their patient flow policies to concord with the risk of infection and ramifications associated with contracting the virus [2,3]. The emergency department managers had to modify their patient flow policies to deal with the threat of people who may have contracted the SARS-CoV-2, an illness known to be with a high infection rate. In this unprecedented and challenging time, emergency department managers had to change the patient flow operations to adhere to governmental regulations such as social distancing, minimizing personal contact, utilizing personal protective equipment, extra sanitary measures, as well as other organization-specific guidelines [4,5].

The COVID-19 virus, recognized for its rapid mutation rate [6], high infection rate [7], and potential severity in immunocompromised patients [8], has highlighted the importance of proactive threat assessment and analysis, ideally achieving this with maximum flexibility, minimal risk, and cost-effectiveness. The COVID-19 pandemic has renewed focus on patient flow within emergency departments, requiring reconsidering clinical patient management strategies [8,9,10].

Hospital emergency departments represent multifaceted systems characterized by numerous dynamic parameters, which make the task of assessing the comprehensive and direct impacts of patient flow modifications quite challenging [11,12,13]. As a result, the application of low-risk, cost-effective strategies, such as computerized simulation modeling, becomes advantageous to assess the potential effects of interventions aimed at mitigating the repercussions of health crises, including the COVID-19 pandemic. The research literature points to evidence that suggests that computerized simulation models provide a resource-efficient approach to evaluating and predicting the consequences of various interventions and modifications on patient flow within healthcare settings [14,15,16,17,18].

The emergency department patient flow issue has previously been investigated through various approaches in the research literature, such as field experiments, surveys, and simulations. An approach to simulation known as “in-situ” implies healthcare workers physically taking part in the simulation [19]. Thus, this methodology represents a considerable requirement both on time and resources, which is increasingly scarce in the healthcare sector. As a more cost-effective and low-risk approach, computer-based simulation modeling has become a more prevalent and attractive alternative [19,20]. The research disclosed in this paper is of the latter category. To improve overall performance, simulation modeling is increasingly necessary for emergency departments to predict what effects changes such as new layouts, new policies, and technologies will have without actually having to implement them [21].

Salmon et al. examined the computerized simulation models applied to emergency department concerns and identified the typical patterns and evolving trends. The review determined that the discrete-event approach was most prominent when examining the performance of processes, resource capacity, and workforce planning within emergency departments [22]. In addition, this finding was corroborated by Hamza et al. [23], who discovered that few simulation models have probed operational patient throughput. Salmon et al. further highlighted that hybrid simulation modeling, i.e., using a combination of modeling approaches (see Section 2.1, p. 3, for a definition of this term), is limited and takes on a more strategic perspective [20]. Nevertheless, hybrid modeling has tremendous potential regarding operational aspects, as demonstrated by Hamza et al., who combined discrete-event and agent-based modeling to simulate the operational patient throughput [22]. On the same note, Friesen et al. have identified agent-based modeling as a simulation modeling paradigm with ‘tremendous potential’ while also pointing out the necessity to combine with other paradigms to construct an accurate simulation model [24].

Previous studies investigated the effect of different intervention policies, e.g., the number of treatment rooms and waiting zones, on several performance indicators (average length of stay, crowding, treatment time) at a specific influx rate of patients that have contracted SARS-CoV-2. Therefore, the purpose of this study is to investigate the effect of patient infection rate on the patient flow by seeking answers to the following two research questions (RQs):

1. What is the likely impact of increased patient infection rate on patient flow parameters in an emergency department?
2. What is the disparate effect of the increased patient infection rate on different COVID-19 intervention configurations in the emergency department?

As indicated by the research questions, this study aimed to investigate the direct outcomes of increased patient infection rate on emergency department patient flow and evaluate the disparate impact on a set of different resource configurations imposed to facilitate the situation imposed by the COVID-19 pandemic in the emergency department.

Concluding this introductory section, Section 1, which has provided background, motivation, context, and the research questions of this study, the rest of the manuscript is portioned into the following three sections. Section 2 lays out the theoretical framework this study builds upon, describes the data types, the emergency department patient flow interventions, and performance indicators, and thoroughly presents this study’s experimental design. The third section, Section 3, of this manuscript gives a combined result and discussion for each of the simulation scenarios run during the execution of this study. The final section, Section 4, of this manuscript will end with a summary of the findings as well as providing the concluding remarks based on the findings of this study.

## 2. Materials and Methods

This section is divided into the following five subsections. First, Section 2.1 presents the theoretical framework this present computerized simulation study is founded and builds upon, as well as delimiting the conceptual scope of its work. Secondly, Section 2.2 presents the different data types used in the computerized simulation study. The next part, Section 2.3, presents the particular case emergency department to lay out and explain the situational-specific circumstances surrounding the case emergency department and present the added resources and interventions subject to the simulation study. The penultimate subsection, Section 2.4, details the patient flow performance indicators used to operationalize the study and elaborates on how those are calculated within the model. Lastly, the final subsection, Section 2.5, details the experimental design of the computerized simulation model and lists the scenarios the study ran.

### 2.1. Theoretical Framework of the Simulation Modeling Method

**Figure 1:**
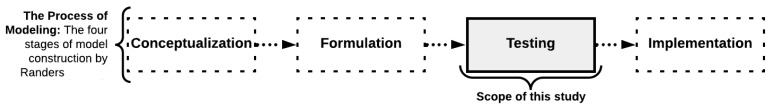
This study’s conceptual scope relation Randers’ four-step simulation methodology [26,27,28]. Figure adapted and reused from previous work [25,29].

As expounded upon in our previous research article [25], we have used agent-based hybrid modeling for this case study, explaining that the workings of Randers give the most appropriate paradigm. As presented in the original literature [26,27,28], the modeling process is a four-step process starting with conceptualization, followed by formulation, testing, and implementation, rendered in the illustration below, Figure 1, adapted and reused from previous work [25,29].

The model that has undergone the above-introduced conceptualization- and formulation stage is a hybrid simulation model that combines the two paradigms of discrete-event simulation modeling (often referred to as DES) and agent-based simulation modeling (often referred to as ABM). A hybrid simulation model, also called a multi-method model, in this work refers to the combination of two or more computer simulation paradigms, as detailed in the workings of Borshchev [30] as well as the more recent works of Ören et al. [31]. This particular combination of simulation modeling paradigms, DES and ABM, was found to be required to achieve to fully capture the full logic of the pandemic-induced interventions onto the patient flow logic within the case organization. This particular combination of paradigms was shown to provide sufficient versatility to reproduce the patient flow logic of the case emergency department operation, especially regarding the prioritization of patient agent groups and which will be expounded upon in subsequent subsections [32].

### 2.2. Types of Data Utilized in This Study

**Figure 2:**
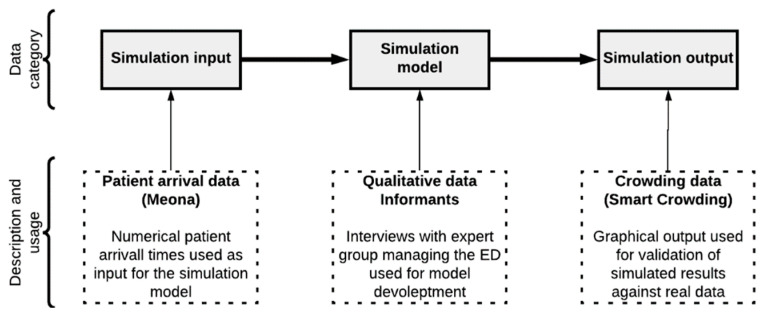
Scheme systemizing the three major datatypes’ relation to the developed model. This figure is reused from previous work [25,29].

The present research disclosed in this paper is based on a computerized simulation model constructed and developed upon three major portions of data as systemized in Figure 2. This figure is reused from previous work [25,29]. The following paragraphs will detail how these data portions were collected, their overall qualities, and how they were implemented to inform this computerized simulation model study.

#### 2.2.1. Patient Influx Data

**Figure 3:**
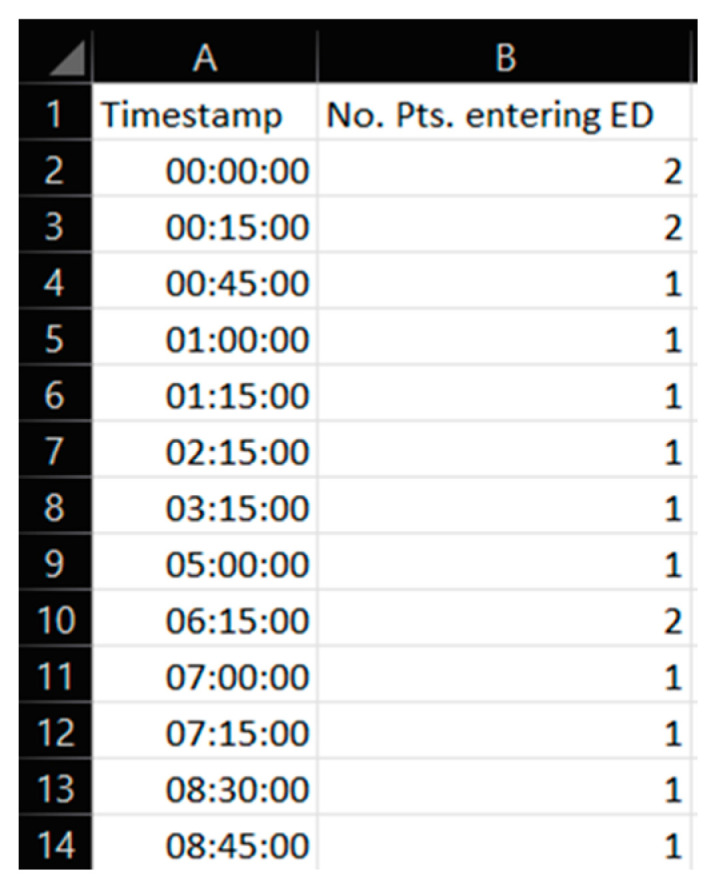
Excerpt from a portion of the numerical data in a spreadsheet used for this study. This data is reused from previous work [29].

The simulation model input uses data from a local database called Meona at the case organization, which, among other types of data not used in this study, keeps a record of the number of patients that have been admitted into the emergency department. These Meona data, of which an illustrated excerpt is shown in Figure 3, are numerical data representing timestamps of arrival for patients; this data is from previous work [29]. Each timestamp represents a patient agent to be inserted into the computerized simulation model. The dataset comes from two selected working days at the case emergency department, which will expound upon some of their characteristics in the later subsection, Section 2.5—Experimental Design.

#### 2.2.2. Qualitative Data

The simulation model itself has been constructed on the basis of knowledge and insights retrieved from expert stakeholders working daily at the case hospital in which this research is concerned. This means that the qualitative data portion mostly lies within the logic and layout of the computerized model itself and has served as the basis for the simulation model conceptualization and formulation. The qualitative data have been gathered through several interviews, meeting discussions, and by walking through the patient flow pathway and logic in the case of emergency department localities. The model development process with specific tools used is disclosed, explained, and related to the theoretical underpinnings of the simulation modeling method in previous works [25,32].

#### 2.2.3. Crowding Data

Computational simulation model output, prior to any of the experimental runs, has been cross-checked with actual crowding curves and systems experts for model verification and model validation. This verification is presented in the previous work [32] and also served the purpose of gaining stakeholder trust and confidence in the model working as it indicated the model performance relative to the real system.

### 2.3. Case Emergency Department, Interventions, and Resources

The case emergency department belongs to a large hospital in the southwest part of Norway. Stavanger University Hospital (SUS) covers the specialized healthcare need of the public in an area encompassing a population of 369,000 [33]. The hospital employs 7800 employees in total. Like most hospitals in Norway, SUS is a publicly funded hospital that is steadily and increasingly subject to financial cuts, fiscal tightening to health care budgets, and limited availability to those scarce resources. The hospital’s emergency department reports that they admit approximately 35,000 patients on an annual basis [34].

The COVID-19 pandemic in Norway resulted in the case emergency department managers to introduce a package of interventions affecting the patient flow process in the department. These interventions were introduced to lower the risk of cross-infection between patients residing within the emergency department. Mere policy changes enabled some of the interventions, while others were enabled by adding physical resources to the emergency department. Table 1 lists and describes the different resources added to the emergency department to target a lower risk of cross-infection within the emergency department.

**Figure 4:**
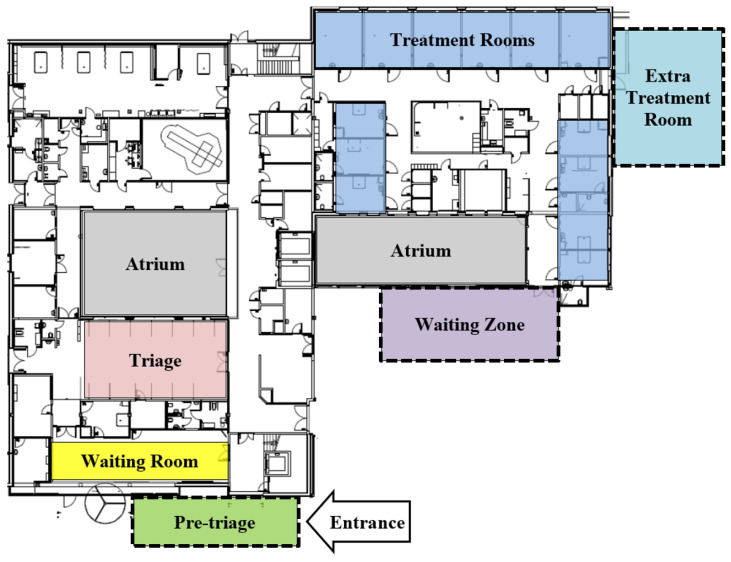
The layout of the case emergency department highlights the new intervention locations. This data is reused from previous work [25,29].

Figure 4 depicts the case emergency department layout and highlights the different resources with colors; this data is from previous work [25,29]. The resources with dotted lines are the resources implemented due to the onset of the COVID-19 pandemic and are used to allow for policies likely to reduce the risk of intradepartmental cross-infection.

The Case Emergency Department’s Patient Flow: Under ordinary circumstances, patients will typically enter the entrance of the emergency department, and once entered, patients will find themselves in a pre-triage. The pre-triage was, at the case organization, introduced as an outdoor installation to the emergency department at the onset of the pandemic. Here, the patient will undergo a patient classification, which here is a specific screening consistent of a questionnaire and antigen test to assess whether the incoming patient should be regarded as infected by COVID-19 (hereinafter denoted to as COVID+) or not infected by COVID-19 (hereinafter denoted to as COVID−).

From this point, the patient flow pathway forward depends on the above-mentioned patient classification. In the case that the individual patient is to be regarded as COVID+ status or COVID−. For the patient that is found to be COVID−, they will have to enter the waiting room as their second local. Once the patient is registered as arrived in the waiting room, the patient can, if there are sufficient resources available, either get a treatment room—if one is available—or go to the triage if there are no treatment rooms available and continue their waiting time in the triage before getting to enter a treatment room where their treatment finally can start. If patients in the pre-triage are found to be COVID+, they will have to immediately be fast-tracked to a treatment room. This fast-tracking is to ensure and reduce the probability of infecting other patients residing within the emergency department. From here, their treatment will start once the proper resources are ready.

Depending on how long patients have been receiving treatment, a COVID− patient may have to leave the treatment room to give up the room for a patient categorized as COVID+ that will need the room, for the above-mentioned reason, to obtain prompt isolation. This will only happen if and only if all of the following four conditions are simultaneously true: (1) if all other treatment rooms are occupied, (2) if the patient has received treatment for at least one hour, (3) if the patient in question is the patient that has received treatment for the longest and (4) if the patient in question is classified as COVID−; in such a special case, the patient in question will have to leave the treatment room and continue their stay in the Waiting Zone. Here, the patient in question has to wait until a treatment room again is available in order to proceed with the necessary treatment before proceeding to a discharge and thereby be able to exit the emergency department.

In the opposite case, if the patient does get categorized as COVID+, the patient will go from the pre-triage and be fast-tracked to a treatment room for immediate isolation. Upon arrival to the treatment room, the patient will start receiving the treatment while healthcare workers follow the strict and appropriate regimen to avoid cross-infection between persons. After receiving the treatment, the COVID+ patient will exit the emergency department and be discharged from the hospital.

Three main resources were introduced to the case emergency department. Table 1 below, adapted from previous work [29], describes and summarizes these three resources under focus. These resources were introduced as intervening measures to the emergency department to combat the adverse risks associated with the pandemic situation, which has been included in the computerized simulation developed for the present study.

### 2.4. Patient Flow Performance Indicators

We incorporated several performance indicators into the model to benchmark the performance of the different scenarios in the study. The majority of them were indicators generally used within the emergency department patient flow studies, while others were introduced in this study aimed to capture the specific effects associated with the introduction of the pandemic interventions. Table 2 below lists the performance indicators and briefly describes them. The following paragraph will provide an in-depth explanation of the patient flow performance indicators ‘average length of stay’ (ALOS) and ‘crowding’, which are of main interest for the result of this paper.

#### 2.4.1. Patient Flow Performance Indicators at Focus

In this present study, there have been two patient flow performance indicators that have been allotted special attention for investigation of the effect of increased patient infection rate on emergency department patient flow: the average length of stay (ALOS) and the crowding.

#### 2.4.2. Explanation and Model Implementation of Average Length of Stay (ALOS)

The average length of stay is perhaps the most widely known and common patient flow performance indicator in the overall literature of patient flow studies. The following equation (Equation (1)), brought from previous workings [32], shows the mathematical logic behind the calculation of ALOS within the computerized simulation model where the patient flow performance indicator is tracked at every timestamp—t in the model run time. ALOS in this work is given in the units of [h/pt], meaning hours per patient. As the equation eludes, at every instance a patient gets discharged from the emergency department, a new patient agent is added to the summarization factor of the equation. ALOS(t) sums all the patient agents staying time (i.e., $P_{\mathrm{Time}\;\mathrm{leaving}\;\mathrm{ED}}\left(n\right)-P_{\mathrm{Time}\;\mathrm{entering}\;\mathrm{ED}}\left(n\right)$) and factors it with the $1/N_{\mathrm{out}}\left(t\right)$ to get the average across the given patient agent population.

(1) $$\begin{array}{c} \mathrm{ALOS}\left(t\right)=\frac{1}{N_{\mathrm{out}}\left(t\right)}\cdot \overset{N_{\mathrm{out}}\left(t\right)}{\underset{n\;=0}{\sum }}\left(P_{\mathrm{Time}\;\mathrm{leaving}\;\mathrm{ED}}\left(n\right)-P_{\mathrm{Time}\;\mathrm{entering}\;\mathrm{ED}}\left(n\right)\right)\\ \forall \;P\in \left[P\;\left(0\right),\;\ldots ,\;P\left(N_{\mathrm{out}}\left(t\right)\right)\right] \end{array}$$

P(n)—nth patient agent, $P_{x}\left(n\right)$—parameter x of the nth patient agent, ${\;N}_{\mathrm{in}}\left(t\right)$—number of patients who entered the ED at time t, ${\;N}_{\mathrm{out}}\left(t\right)$—number of patients left the ED at time t, t—time; acting as the independent discrete time variable going from 00:00:00 to 23:59:59, running in increments of seconds.

The average length of stay is calculated for the aggregate total group of patient agents (Tot.), the patients classified with the COVID+ status, and the patients classified with the COVID− status. Hence the equation within the computerized simulation model is realized in serving the measurement of ALOS for the three different patient agent populations.

#### 2.4.3. Explanation and Model Implementation of Crowding

In this study, the crowding measure was designed to reflect the case organization implemented a framework of hospital-wide patient flow called high patient prevalence under the name ‘plan for high activity.’ Thus, the operationalization and naming is from the case organization itself. The mentioned patient flow improvement framework pointed out three crucial levels of patient prevalence; 15, 25, and 30. Each of these levels has its specified package of initiatives to alleviate the higher crowding of patients.

In the computerized simulation model, the crowding performance indicator takes the outset in the number of concurrent patients described in the following difference of the number of patients that have been admitted to less the number of patients that have been discharged, giving the following difference at any time—t : $N_{\mathrm{out}}\left(t\right)-N_{\mathrm{in}}\left(t\right)$. A simple if-statement keeps track of the difference and yields one if the difference is higher than the three levels mentioned above of 15, 25, and 30 patient agents. Based on this function, denoted by u(t), each timestamp of the model run-time gets summed towards the current value in run-time. The sum is divided by the total run-time and thus yields a number between 0 and 1, which in turn is scaled to a 0–100% scale for presentational purposes.

(2) $$\begin{array}{c} {\mathrm{Crowding}}_{C}\left(t\right)=\frac{\sum_{n=0}^{t}u\left(t\right)}{t}\;\cdot 100\%,\;u\left(t\right)=\{\begin{array}{c} 1\;\mathrm{if}\;\left(N_{\mathrm{out}}\left(t\right)-N_{\mathrm{in}}\left(t\right)\right)>C\\ 0\;\mathrm{otherwise} \end{array}\\ C\in 15,\;25\;\&\;30 \end{array}$$

P(n)—nth patient agent, $P_{x}\left(n\right)$—parameter x of the nth patient agent, $N_{\mathrm{in}}\left(t\right)$—number of patients who entered the ED at time t,${\;N}_{\mathrm{out}}\left(t\right)$—number of patients left the ED at time t, t—time; acting as the independent discrete time variable going from 00:00:00 to 23:59:59, running in increments of seconds.

### 2.5. Experimental Design and Simulation Model Scenarios

This section aims to show the experimental decisions made during the development of the computerized simulation model utilized in this study. Overall the present study’s experimental design closely follows that of a previously published article within this research endeavor [29], except that this study focuses on the patient flow performance with increasing rates of infection among the admitted patients. This means that the different simulation runs vary in one parameter of particular interest: the patient infection rate (PIR). The reported result shows the performance of the emergency department patient flow performance indicators with increased PIR. In contrast to the performance indicators presented in the previous subsection, Section 2.4, the PIR is a constant, meaning it is time-invariant within each simulation run of the model although is incremented, in the range from 0% to 50%, from one simulation to the next. Below is the equation, Equation (3), describing the operationalization PIR into the simulation model of this study.

(3) $$\mathrm{PIR}=\;\frac{N_{in}^{COVID+}}{N_{in\;Tot.}}\;\%,\;\;\;\;\;\;\;\;\mathrm{PIR}\in \left[0,\;5,\;50\right]\%$$

PIR—patient infection rate [%], $N_{\mathrm{in}}^{\mathrm{COVID}+}$—number of patient agents inserted to the model assigned with the COVID19+ status, $N_{\mathrm{in}\;\mathrm{Tot}.}$—the total number of patient agents inserted into the model during the total runtime of the simulation, PIR∈[0, 5, 50] %—denotes an array of values, with the PIR taking a range of values from 0% to 50% with 5 percentage points of increments, yielding 11 simulation runs for each scenario.

This study targets to evaluate the differential outcomes of the patient flow metrics during increased PIR. As we are in here studying the impact of different PIRs on the patient flow in the ED, the PIR parameter plays a central role in this study; we will here go into detail on the underlying mechanism of this parameter in the computerized simulation model. Within the simulation model, PIR is the parameter within the computerized dictating which proportion of patients are found to be COVID+ in the pre-triage. During the run-time of the simulation, patient agents will arrive according to the list of patients that we have described in the previous subsection, Section 2.2—Types of Data Utilized in Study. In the pre-triage, patient agents will be categorized as COVID+ at a rate of PIR. Conversely, patients will be categorized as COVID− at a rate of 1 − PIR. The PIR thus represents the probability for each patient to be categorized as COVID+.

To accomplish the experimental need of this study, we had to go through the simulation model to ensure the scenario ran with the supposed PIR. This was achieved by making the patient agent classification a self-regulating process depending on the scenario’s pre-determined level of patient infection rate. Suppose the actual patient infection rate is less than the nominal patient infection rate. In that case, the next patient agent incoming the model will be classified as COVID+ and vice versa. This control mechanism thus makes the patient infection rate non-stochastic. Equation (4) illustrates how the logic is programmatically carried out by a simple if-statement on each newly injected patient agent into the simulation model.

(4) $$\mathrm{Patient}\;\mathrm{agent}\;\mathrm{status}=\{\begin{array}{c} \mathrm{COVID}+\;\;\;\;\;if\;\;\;\;\;\mathrm{PIR}\;\leq \;\frac{N_{in}^{COVID+}}{N_{in\;Tot.}}\\ \mathrm{COVID}-\;\;\;\;\;\;\;\;\;\;\;\;\;\;\;\;\;\;\;\mathrm{otherwise} \end{array},\;R\in \left[0,\;5,\;50\right]\%$$

PIR—patient infection rate [%], $N_{\mathrm{in}}^{\mathrm{COVID}+}$—number of patient agents inserted to the model assigned with the COVID19+ status., $N_{\mathrm{in}\;\mathrm{Tot}.}$—the total number of patient agents inserted into the model during the total runtime of the simulation, PIR∈[0, 5, 50] %—denotes an array of values, with the PIR taking a range of values from 0% to 50% with 5 percentage points of increments, yielding 11 simulation runs for each scenario.

Thus, as will be presented in the results, Section 3—Results and Discussion, some of the mentioned patient flow performance indicators will be given distinctly for the group of patient agents that are categorized as SARS-CoV-2 virus infected (COVID+), those who are not categorized as infected (COVID−), and lastly, in order to track the overall patient performance, there is a combined category group as the whole, i.e., combining both of the patient groups. Table 3 summarizes these patient agent groups.

The next issue of importance with regards to the experimental design of the conducted study is the sampled days that the patient influx data are gathered from. There are two days represented in the study. In the study, we look at individual days rather than the average of them; this was an explicit choice made to ensure that we could see the patient flow characteristics of two representative days and not the mere average that possibly could masquerade much insightful information particular to those different days, e.g., depending on the influx profile, a parameter such as a peak crowding (explained in Table 2) would be reduced and thus not give the full picture of how a high-demand day would take a toll on the emergency department resources.

**Figure 5:**
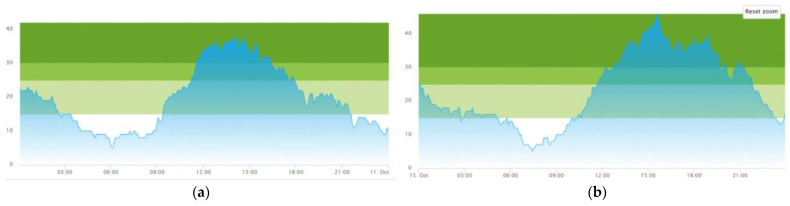
Graphs of the patient crowding at the emergency department for the two days of this study. The coloring in graph represents the three different Crowding levels as presented in Equation (2) of Section 2.4.3. ‘Explanation and Model Implementation of Crowding’: Light Green; Crowding ≥ 15, Green; Crowding ≥ 25, Dark Green; Crowding ≥ 30 (**a**) Day 1—‘Average patient influx day’, with a close to average patient influx at 104 patients. (**b**) Day 2—‘High patient influx day’, with higher than average patient influx at 121 patients. This data is reused from previous work [25,29].

Two representative days were chosen in this study, Day 1—‘Average patient influx day’, and Day 2—‘High patient influx day’. The first day, Day 1, overall is a day where the demand is at a high pace, although not exceeding what is regarded to be normal, leading the emergency department to pass the 30-patient crowding threshold spiking around 2 o’clock with a patient crowding of 37 patients. Although the emergency department this day operates with a generally high crowding, this day is not unusual for the case emergency department. The total amount of patients admitted to the emergency department on this day comes to 104 patients, which is reasonably close to the daily average for this case emergency department at 100 patients. The second day (Day 2) is a day of an even higher pace of patient influx. As discovered in the results chapter for this paper, Section 3—Results and Discussion, there is a mismatch between the capacity and the demand on this day, yielding a higher patient prevalence throughout this day. Patients admitted this day are totaling 121 which, in contrast to Day 1, is substantially above the daily average of 100 admitted patients a day. Figure 5 shows graphical data from previous work [25,29] on the two days of the case emergency department internal performance metric ‘crowding’, a patient flow performance indicator previously defined in Table 2.

The last major point of the experimental design of this study is the model resource configurations. At the onset of the pandemic, the emergency department managers added a set of rules for the patient flow as well as two portions of physical resources to the emergency department; a dedicated waiting zone (WZ) and a set of extra treatment rooms (E.TR.). We have divided configurations into four different scenarios, where the first scenario (Sc. 1) constitutes the emergency department resources including those prior to the pandemic situation. Following is the second scenario (Sc. 2), which adds the waiting zone (WZ) to the emergency department (please refer to illustrated resources in the blueprint of Figure 4). Scenario 3 (Sc. 3) does not include the waiting zone but introduces the extra treatment rooms (E.TR.). Lastly, the final scenario (Sc. 4) combines the resources of Sc. 2 and Sc. 3, as this runs with both the waiting zone and the extra treatment rooms (WZ and E.TR.) included in the simulation model. To address the research aim, to study the impact of increased patient infection rate, in this present study, all the aforementioned scenarios are run in series where the patient infection rate (PIR) starts at 0% and increases with 5 percentage points until the maximum patient infection rate of 50%. Table 4 lists and summarizes the four scenarios conducted in the present study.

These four scenarios were run with a PIR going from 0% to 50% with a 5pp increment yielding 11 simulation runs for each of the seven configurations, as tabulated in Table 4, with the two different days, Day 1—‘Average patient influx day’ and Day 2—‘High patient influx day’, meaning there was ran a total of 154 simulation runs for the results reported in this study. The top PIR limit of 50% was put as a theoretical maximum where any higher patient infection rates very likely would fully alter the approach from the emergency department leaders as this, in practice, would negate most of the utility in segregating the COVID+ and COVID− patients.

## 3. Results and Discussions

Due to this present research’s inseparable nature of the results and the discussion of the results, this section combines both of these elements in one section. This section reports on three groups of results and discusses these along with their respective presentation. First, the average length of stay (ALOS) results for four scenarios (Sc. 1–Sc. 4, as described in Section 2.5—‘Experimental Design and Simulation Model Scenarios’ and Table 4) over several PIR during a day of average patient influx (hereinafter denoted to as ‘Day 1’) and during a day of high patient influx (hereinafter denoted to as ‘Day 2’) conditions are presented, followed by histogram charts that illustrate the variation in the patient agent length of stay (LOS) to explain the fluctuation phenomenon observed in some of the resulting curves. Second, the crowding results for four scenarios over several PIR under Day 1 and Day 2 conditions are presented and discussed. Third, the simulated results as the combined effect of PIR and the number of extra treatment rooms on the ALOS and crowding in Day 1 and Day 2 conditions are illustrated.

In summary, there are seven specific points of discussion: (1) How ALOS develops across several patient infection rates (PIR) for each scenario (no intervention, only introducing waiting zone, only introducing extra treatment rooms, both introducing waiting zone and extra treatment rooms)? (2) How ALOS behaves under Day 1 and Day 2 conditions? (3) How ALOS behaves for COVID− and COVID+ patients? (4) How crowding behaves over several PIR for each scenario? (5) How crowding behaves in Day 1 and Day 2 conditions? (6) How ALOS behaves at a different number of extra treatment rooms? (7) How crowding behaves at a different number of extra treatment rooms?

This results and discussion will limit the presentation and hold focus on two of the patient flow performance indicators, which have been detailed in Section 2.4. The full simulation output across all the scenarios showing every patient flow performance indicator implemented into the computerized simulation model is, however, disclosed within the appendix section, Appendix A, of the present manuscript, from Table A1 to Table A4. The graphs presented hereunder in the current discussion are created on the back of the numerical output in mentioned tables. The following paragraphs will provide this output as well as a thorough analysis of the computerized simulation output yielded from running the scenarios from Sc. 1 to Sc. 4.

### 3.1. Results from Running Scenario 1—No Added Resources

Per the experimental design detailed in Section 2.5—‘Experimental Design and Simulation Model Scenarios’, Scenario 1 (Sc. 1) constitutes the scenario configuration where there are no added resources to the emergency department and no changes other than the introduced patient flow intervention of the prioritization policy, i.e., fast-tracking and isolation, of the COVID+ patients agents in order to reduce the risk of intradepartmental cross-infection. Meaning there is no use of the extra treatment rooms or the waiting zone, which will be tested in the subsequent scenarios.

**Figure 6:**
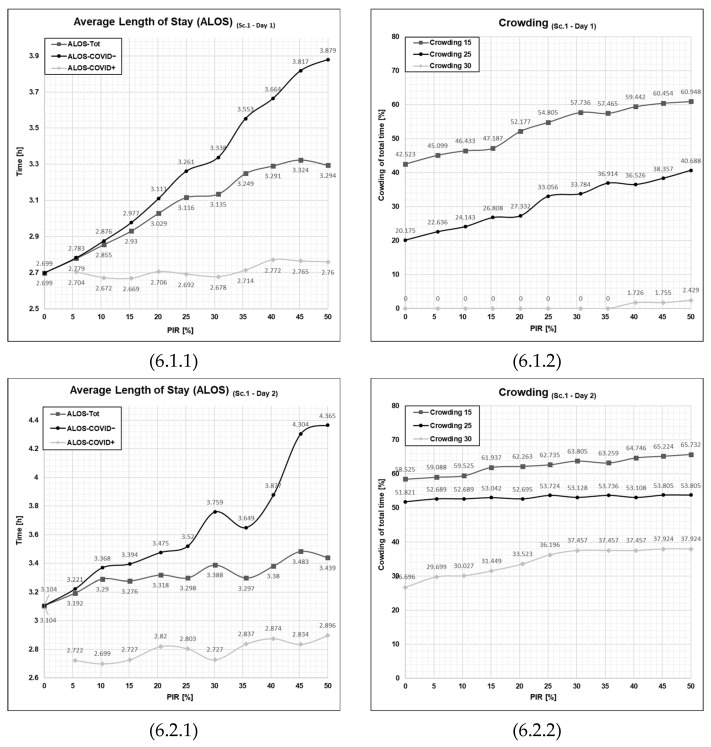
Graphical outputs from simulation run illustrating the impact of increased PIR on both ALOS and crowding in Day 1 and Day 2 in Scenario 1. Sc—scenario, Tot.—total patient population, Ord.—The patient agent group classified as COVID−, Con.—The patient agent group classified as COVID+, PIR—patient infection rate, h—number of hours. Cell references follows the following format (Figure No., Day; 1 = Day 1 or 2 = Day 2, PFPI; 1 = ALOS or 2 = crowding). Numerical values are given for reference in Appendix A in Table A1, Table A2, Table A3 and Table A4.

Initial observation of the scenario output graphs in Figure 6, depicting the differential outcome for the two different patient agent groups, the average length of stay for COVID+ patients (See Figure 6, cell 6.1.1—ALOS-COVID−) is consistently much lower than that of COVID− patients agent in all the different levels of PIR. The difference between the two patient agent groups increases along with the increased level of PIR. The ALOS for COVID+ patients does not increase dramatically with increasing PIR, but ALOS for COVID− patients does increase sharply with increasing PIR. By examining the differences between consecutive values in the ALOS-COVID− patient agents closely, we can observe that the values generally increase as the PIR increases. However, the rate of increase is not consistent throughout the development of PIR, going from 0% to 50%. Initially, in the portion where PIR ranges from 0 to 25%, the development takes on a general convex pattern, meaning that each increment yields an acceleration of ALOS. Towards the end of the curve, PIR at 30% and above, the increase becomes relatively smaller for each increment, and the curve takes into a concave form. The portion PIR at 25% to 30% seems to be a breaking point where the system response breaks from a convex pattern to a concave pattern, yielding a slightly unexpected indent in the graph.

Examining the values for the COVID+ patient agent (See Figure 6, cell 6.1.1—ALOS-COVID+), contrary to the COVID− patient agents, the data here does not show a clear trend or consistent pattern of increase or decrease along with the increments of PIR from 0 to 50%. Instead, the output values for the ALOS-COVID+ vary within a relatively narrow range. There are both portions where the graph increases and decreases, indicating no strong directional movement. By implication for Scenario 1 in Day 1, the throughput for COVID+ patients is consistent, as the patient agent is not stalled from the fast-tracking process.

Looking towards the other patient flow performance indicator, crowding (Figure 6, cell 6.1.2), the results here reflect a similar trend to that of ALOS-COVID−, where we can observe that crowding also increases along with an increased level of PIR. Due to the lower patient influx, we see that the 30% threshold, crowding 30, is not crossed until the patient infection rate hits a level of 40%, and from that point, it is only at very moderate percentages hitting the 2.429% at PIR = 50. Although the crowding 30 represents a critical level of crowding, the output values suggest that this threshold is only reached at very brief moments during the simulated day. The crowding 25 goes from 20.175% at the PIR = 0% to 40.688% at PIR = 50% with no particular pattern other than what is approximately a linear development. The gradual increase corresponds to a 2.05 percent point increase in crowding 25 for each 5 percentage point increment of PIR.

The graphs in the lower half of the second row of Figure 6 show the output from the second day, Day 2, which is, as previously presented, a day of higher patient agent influx to the emergency department. It is apparent that the emergency department is at a far higher occupancy and utilization than on the first day, Day 1. Overall, the graphs show a reduction in the linear development than that of Day 1, which makes sense that the higher patient influx days gives higher variability in patient flow performance.

Looking closer at the data, the average length of stay for the COVID− patient population (Figure 6, cell 6.2.1 ALOS-COVID−) ranges from a 3.104 h at a patient infection rate of 0%, and on the other extreme, at 50%, it reaches 4.465 h, which means that most patients spend a lot more time within the treatment room at the highest patient infection rate. The convex trajectory observed in the Day 1 is not present for the graph for Day 2. The output is overall more volatile and unpredictable.

The second graph for Day 2 (Figure 6, cell 6.2.2) shows the ‘crowding’ (see Section 2.4 for the definition) for the Day 1 of high patient influx. On the contrary, from Day 1 to Day 2, a far more high-pressure situation occurs in the emergency department. The 30% crowding is at the onset of 0% patient infection rate at 27%, which is a high-pressure situation straining the emergency department. With the increased patient infection rate, the 30% crowding approximately plateaus at 30%, which appears to be a saturation point for the patient infection rate on the crowding outcome hitting 37–38% on 30% crowding from this point and onwards.

What is striking by a closer examination of the graphs from Day 1 with the graphs from Day 2, the first and second row in Figure 6, respectively, is the deviancies from a linear development in the output. For Day 1, the deviances are so small that they, under most circumstances, could be written off as deviances within a reasonable margin of error. The deviances on Day 2 were far more pronounced. This could point towards a tendency for the patient flow process in the emergency department turns more non-linear with increased predictability when the patient influx and PIR increase. All in all, by running the first Sc. 1, we see what difference there is between the COVID− patient agent influx and the high patient influx. We see that increased PIR has amplified this difference, especially when the PIR is at the higher end of the scale.

Scenario 1 showed that during a day with a moderate influx of patients, there is only at a 45% patient infection rate level that the emergency department reaches a critical crowding that needs the highest level of attention. What is worth noting is that Scenario 1 is where no resourcing initiatives are being taken to alleviate the situation. That will be investigated in the subsequent scenarios, Sc. 2–Sc. 4, presented in the following subsections.

### 3.2. Results from Running Scenario 2—Added Waiting Zone

Scenario 2 is the series of scenarios where a waiting zone (WZ) is added to the emergency department. In the patient flow operation, the waiting zone will be used under certain circumstances; all the treatment rooms must be occupied, and another COVID+ patient agent must arrive at the emergency department. In this case, the COVID− patient that has spent most of the time in the treatment room but no less than 60 min will be released from the treatment room and go to the waiting zone. In contrast, the incoming COVID+ patient at the pre-screening at the pre-triage will be fast-tracked to the treatment room in order to avoid or minimize the risk of cross-infection with other patients or healthcare workers within the emergency department.

**Figure 7:**
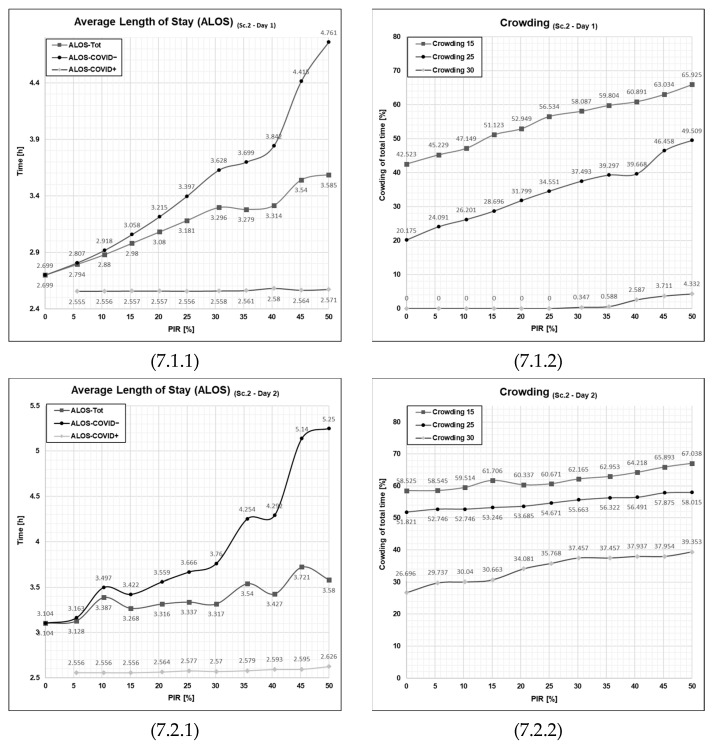
Graphical outputs from simulation run illustrating the impact of increased PIR on both ALOS and crowding on Day 1 and Day 2 in Scenario 2. Sc—scenario, Tot.—total patient population, Ord.—the patient agent group classified as COVID−, Con.—the patient agent group classified as COVID+, PIR—patient infection rate, h—number of hours. Cell references follows the following format (Figure No., Day; 1 = Day 1 or 2 = Day 2, PFPI; 1 = ALOS or 2 = Crowding). Numerical values are given for reference in Appendix A in Table A1, Table A2, Table A3 and Table A4.

Looking at the output graph showing the ALOS for Scenario 2 Day 1 (Figure 7, cell 7.1.1), we observe that the curve in the ALOS-COVID− has the same outset (both start exactly at 2.7 h) at a 0% patient infection rate as that of Scenario 1 (Figure 6, cell 6.1.1). However, the curve in Scenario 2 is steeper, meaning that for each increment (5 percentage points increase in patient infection rate), the average length of stay for the COVID− patient agents (ALOS-COVID−) increases more. This trajectory indicates that the setup constituted by the resource configuration in Scenario 2 is more sensitive to the increased patient infection rate. Overall, the average length of stay for COVID+ patient agents is still much lower than for COVID− patient agents and is also lower than ALOS for COVID+ patients in Scenario 1, Day 1 (Figure 6, cell 6.1.1). However, the improvement for COVID+ patients comes at the expense of COVID− patients. Their ALOS is higher than that of Scenario 1, Day 1, and the disadvantage for COVID− patients relative to COVID+ patients increases with increasing PIR.

When observing the outcome of the crowding performance indicator (See Figure 7, cell 7.1.2), the average length of stay starts at the same point as the previous scenario, Scenario 1, at 0%, 20.2%, and 42.5% for 30%-, 25%-, and 15%-crowding, respectively, for the 0% patient infection rate. This difference has a simple explanation: when the PIR is at 0%, there will not be any patients entering the emergency department that qualifies for the usage of the waiting zone to be utilized; thus, in the case of 0%-PIR, these two scenarios will have the same outcome. However, if we take a closer look at the advancement of patient infection rate, the development, similarly to the ALOS Sc. 1 vs. Sc. 2, is steeper, although we do not see the patient performance indicator plateauing here. In fact, the 15%- and 25%-crowding increases along with the PIR for all the increments of PIR from 0% to 50% patient infection rate. Here, we also see, as in scenario 1 for Day 2, that the 30%-crowding does not appear before the patient infection rate is 40%.

Turning towards the second row of Figure 7 and analyzing the outcome of the high patient influx from Day 2, we unsurprisingly see more stress in the emergency department overall. The average length of stay (Figure 7, cell 7.2.1) is, in this scenario, more unstable as it, even more than for Scenario 1, deviates from a linear development across the 0% to 50% development. In fact, the scale of this graph had to be changed in order to capture the final ALOS for COVID− patients at a 50% patient infection rate, reaching a 5.25 h ALOS-COVID− at the 50% patient infection rate, illustrating the increased burden onto the COVID− patients, whereas the COVID+ patients have a very stable trajectory throughout the increase of PIR from 0% to 50%.

Looking at the crowding patient flow parameter for Day 2, the difference is pronounced when compared to the graph for Day 1. However, more interestingly, the impact is not as drastic as on Day 2 of Scenario 1. Scenario 2 has a peak in crowding at 30% of 39.4% while the same peak is 37.9% attesting to a lesser degree of impact from the added waiting zone compared to the average stay length.

In summary, running Scenario 2 has shown the configuration of resources with the added waiting zone. By examining these two selected patient flow performance indicators, the waiting zone may appear as a net burden for the emergency department patient flow by seriously disaffecting the COVID− patients with a drastically increased average length of stay. Much of this increase naturally is explained by the waiting zone that COVID− patients had to wait in, adding time to their overall stay time. However, even though this initiative has a net reduction of patient flow performance indicators, the aim of implementing the waiting zone is to accomplish a strictly needed service of yielding the possibility of swift fast-tracking for COVID+ patients. It is quite interesting to observe the disparate sensitivity of the two parameters ALOS and crowding, where crowding is less affected by the new configuration constituted by Scenario 2. The average length of stay has a much larger impact, pointing out that the individual patient impact caused by the change is much larger than when looking at the whole department.

### 3.3. Results from Running Scenario 3—Added Extra Treatment Rooms

Scenario 3 corresponds to the resource configuration where there are added extra treatment rooms to the emergency department. The patient flow logic will match that of Scenario 1 as no changes are performed to the patient flow logic, just the fact that the emergency department now contains four more treatment rooms, which will be start used once the regular treatment rooms are fully utilized; thus, the total number of treatment rooms in this series of the simulation run is 17 in total.

**Figure 8:**
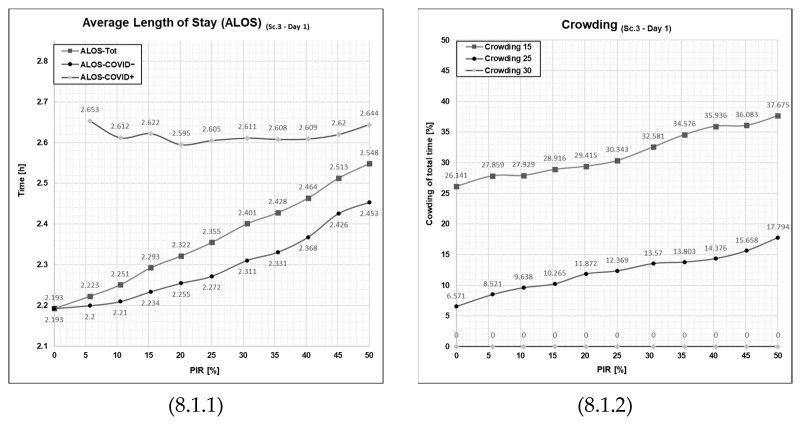

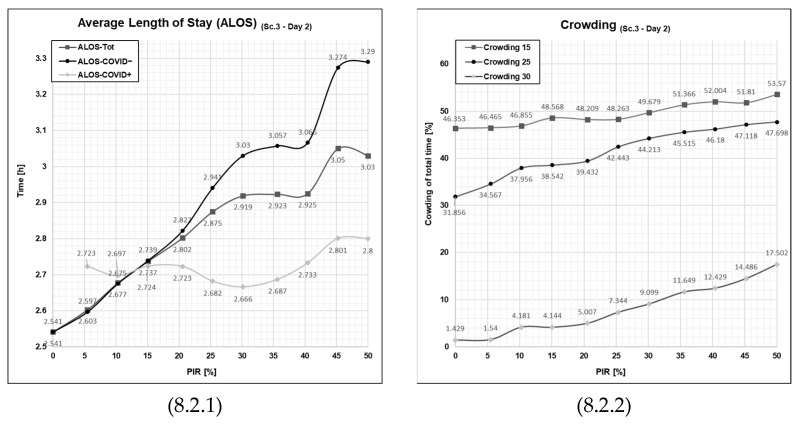
Graphical outputs from the simulation run illustrating the impact of increased PIR on both ALOS and crowding in Day 1 and Day 2 in Scenario 3. Sc—scenario, Tot.—total patient population, Ord.—the patient agent group classified as COVID−, Con.—the patient agent group classified as COVID+, PIR—patient infection rate, h—number of hours. Cell references follow the following format (Figure No., Day; 1 = Day 1 or 2 = Day 2, PFPI; 1 = ALOS or 2 = crowding). Numerical values are given for reference in Appendix A in Table A1, Table A2, Table A3 and Table A4.

When looking at Day 1 for ALOS (Figure 8, cell 8.1.1), the results are very favorable in this scenario for both the COVID− and COVID+ patient groups. ALOS for COVID− patients registers even lower than for COVID+ patients within the full scale of patient infection rate from 5% to 50%. This is contrary to what has been the previous results yielded in both Scenario 1 and Scenario 2. The most remarkable feature of this scenario is the flatness of the curves: ALOS for both patient groups remains at a stable low level, even at increasing levels of PIR. Hence, this intervention seems effective in that the ALOS is far less sensitive to increases in patient infection rates.

The same can be said for the crowding indicator as this is also much lower in this scenario compared to the two previous ones, Scenario 1 and Scenario 2. The sensitivity towards the patient infection rate is lower for crowding 15 and crowding 20. Even more notably, the patient flow indicator crowding 30 here is completely flat at 0% at every rate from 0% to 50% of patient infection rates, meaning a much more favorable situation for the emergency department.

Another observation to point out in Scenario 3, Day 2 (Figure 8, cell 8.2.1) is that ALOS for COVID+ is remarkably stable on a fairly low level even with an increase of PIR (as in Day 1—‘Average patient influx day’). For COVID− patients, however, ALOS is higher than on the day of average patient influx (Figure 8, cell 8.2.1) and upholds the regular pattern found in the other outputs that it rises along with increasing patient infection rate. Still, ALOS, even for COVID− patients, remains lower than in Scenarios 1 and 2 for the higher patient influx day (Day 2).

Crowding is also higher on the day of high patient influx for Scenario 3 (Figure 8, cell 8.2.2) compared to Day 1, and we see here that the patient infection rates over 5% make the emergency department reach the critical crowding 30 threshold, and at higher patient infection rates, it increases and results on the final value of 17.5% at a 50% patient infection rate, which is characterized as a high-intensity day but far lesser than the level observed in the previous scenario.

A possible explanation for this observed difference between Day 1 and Day 2 might be found in the fact that the data representing the influx of patients on Day 2 is higher (20%) than on Day 1. A way to understand the results can be that if the influx (as in Day 1) is within the available capacity of the emergency department that day (including the added four extra treatment rooms), then the ED is able to manage the patient flow well with low levels of crowding (as seen in Figure 5, crowding in Sc. 1, Day 1), even at high levels of PIR. However, if the level of patient influx (as on Day 2) goes over the capacity of the emergency department, it will lead to more chaotic, non-linear behavior, higher levels of crowding, and a higher level of ALOS for COVID− patients, rising with increasing PIR. However, even on the day of high patient influx (Day 2), in Scenario 3 (introducing extra waiting rooms), ALOS levels for COVID− patients are still lower than in Scenarios 1 (no added resources) or Scenario 2 (waiting zone) at comparable PIR levels. In addition, even with the increased patient influx as on Day 2, ALOS for COVID+ patients remains at a fairly stable level even with increasing PIR.

### 3.4. Results from Running Scenario 4—Added Waiting Zone and Extra Treatment

Scenario 4 denotes the series of simulation runs, which combines the efforts from Scenario 2 with the introduced waiting zone and Scenario 3 with the introduced extra treatment rooms in order to measure the combined effect of those two interventions. Thus, the patient flow logic is a combination of that of Scenario 1 and Scenario 2.

**Figure 9:**
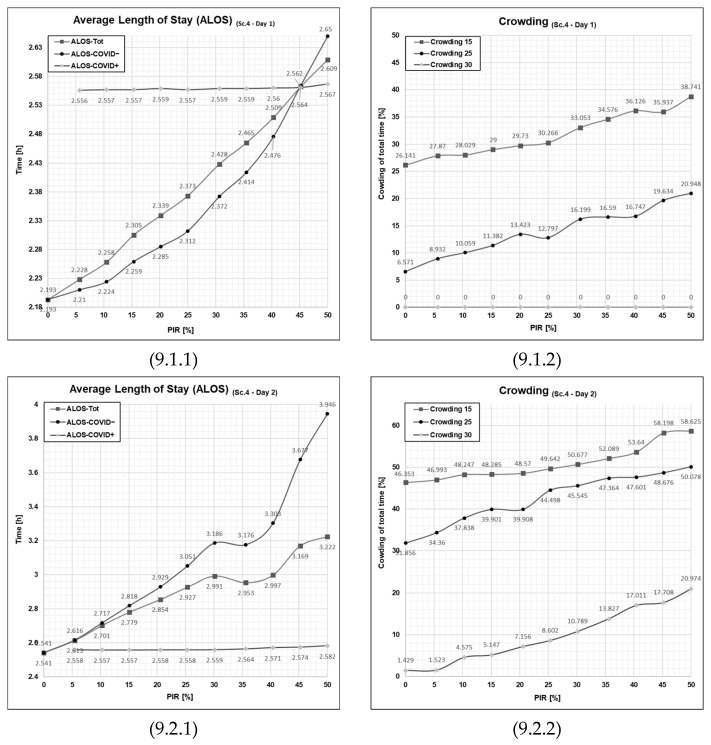
Graphical outputs from the simulation run illustrate the impact of increased PIR on both ALOS and crowding in Day 1 and Day 2 in Scenario 4. Sc—scenario, Tot.—total patient population, Ord.—the patient agent group classified as COVID−, Con.—the patient agent group classified as COVID+, PIR—patient infection rate, h—number of hours. Cell references follows the following format (Figure No., Day; 1 = Day 1 or 2= Day 2, PFPI; 1 = ALOS or 2 = crowding). Numerical values are given for reference in Appendix A in Table A1, Table A2, Table A3 and Table A4.

By running the series of simulation runs, we see that the average length of stay during the day of moderate patient influx (Figure 9, cell 9.1.1), for the COVID+ patient, is even more stabilized but overall exerts only minor changes from that of Scenario 2 and 3. While for COVID− patients, the average length of stay exerts a minor increase compared to Scenario 3. However, the change only to be visually recognizable in the higher patient infection rates of 30% and above. This, of course, is explained by the fact that the waiting zone for COVID− patients constitutes an additional delay point for certain subsets of patients.

Referring to the simulation output for crowding in for Day 1 in Scenario 4 (Figure 9, cell 9.1.2), we see a graph that, for all intents and purposes, is identical to that for Day 1 Scenario 3 (Figure 8, cell 8.1.2). Thus, by implication, compared to Scenario 2 (Figure 7, cell 7.1.2), we see the same overall improvements compared to the base case Scenario 1.

More interesting is to observe the effect of the combined contribution of the waiting zone and the extra treatment rooms when looking at a higher intensity, Day 2 (Figure 8, cell 8.2.1). Taking a look at the average length of stay, we see that compared the scenario 3 (Figure 7, cell 7.2.1), the ALOS-COVID+ has gotten stabilized onto its theoretic minima, which is great. This means that no COVID+ patients had to wait before getting fast-tracked to an available treatment room. The other side of this coin, however, is that we see this comes at some cost of an increased average length of stay for the COVID− patients, which has a steep trajectory across the increased patient infection rates.

Throughout the runs of Scenario 4 on Day 2, we again see that the crowding holds equal to Scenario 3; here, if we consider the fact that the emergency department overall can store six more patients when taking into account the full capacity of the waiting zone, this means that a slight increase compared is expected.

Seeing the result from Scenario 4 and comparing it to the previous result from Scenario 3, one may, by looking at Day 1 isolated, conclude that the patient flow operation is indistinguishable from each other in terms of the average length of stay and crowding across the variety of patient infection rates. However, this holds true because we are looking at a configuration of the emergency department that is well-balanced with the demand of this given day with the lower patient influx. What also needs to be taken into consideration is when comparing to Scenario 3, even though the parameters develop almost identically across the increase of patient infection rate, one must carry in mind that the emergency department can hold six more patients in productive position meaning that the equal output means that Scenario 4 yields a better score than Scenario 3.

Upon evaluating Day 2, which experienced a greater patient influx, subtle overall changes were observed. The emergency department demonstrated an improved capacity to manage the heightened patient volume, albeit at the expense of COVID− patients. A significant advantage of this scenario is the stable throughput of COVID+ patients, a substantial improvement given the potential severity of cross-infection within emergency department settings.

#### Discussing the Observed Non-Linearities in Sc. 1–4

**Figure 10:**
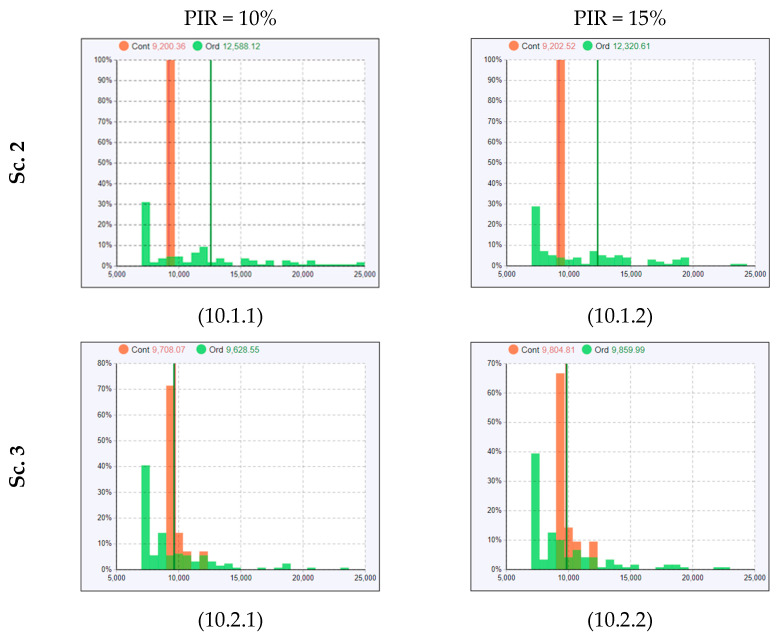
Histogram displaying the length of stay frequency across the patient agent populations.

In Figure 7, Figure 8 and Figure 9, the ALOS curves for Scenario 1–4 (particularly cells 6.1.1, 6.2.1, 7.2.1, 8.2.1, and 9.2.1) show similar overall trends as discussed in the conditions of average patient influx (Day 1), but with a non-linear behavior (fluctuation where ALOS for PIR = 15% is lower than ALOS for PIR = 10%). The average length of stay for COVID− patients at PIR% for Scenario 2 on Day 2 is about 3.5 h. However, it is 3.4 h at PIR = 15%, where we should expect higher rather than lower ALOS. Let us look into the histogram of the length of stay and observe why the average value has such fluctuation. Histograms are an effective tool for identifying the shape of the distribution. The shape of the distribution is a fundamental characteristic of the sample that can determine which measure of central tendency best reflects the center of the data. In Figure 10, the histograms for Scenario 2 (Figure 10, cell 10.1.1 and cell 10.1.2) show multinomial distribution, where there are more than two outcomes. For example, in our case, some patient agents stay 10,000 s, others stay 12,000 s, others stay 15,000 s, and others stay 20,000 s. Thus, taking the average for a multinomial histogram is ineffective for a central tendency.

Understandably, COVID− patients (represented with the green bars in the histogram) might have a different average length of stay, as some COVID− patients stay in both treatment rooms and the waiting zone, and some wait only in treatment rooms. In Scenario 3 (Figure 10, cell 10.2.1 and cell 10.2.2), where more treatment rooms were utilized, the length of stay histograms have a better distribution (almost binominal) as offering more rooms reduces the stay time in the waiting zone. Thus, taking the average in Scenario 3 is more effective in representing the central tendency and tracking the change over several PIR-rates.

Our findings above confirm the findings in a previous study [29] that the intervention of implementing the waiting zone is the most effective for COVID+ patients, but may not be the most effective intervention in total, given that it negatively affects the patient flow of COVID− patients. What we now also see in the current study is that this negative effect worsens with increasing PIR. The introduction of extra treatment rooms, however, leads to a beneficial effect both for COVID+ and COVID− patients, as we saw [29], but the new finding in the current study is that this beneficial effect is less dependent on PIR—provided that the patient influx stays within the capacity of the emergency department. Thus far, the intervention of “extra treatment rooms” we had introduced in our simulation was set at four extra treatment rooms. As our simulation results above show, four extra treatment rooms were sufficient to create an ED setup where ALOS was fairly insensitive to increasing PIR on a day with average patient influx (Day 1), but not on a day of high patient influx (Day 2).

### 3.5. The Effect of Extra Treatment Rooms on the ALOS and Crowding

A relevant question that thus arises is: How many extra treatment rooms does one need to introduce to obtain the best benefit of this intervention? To pursue this question, we decided to run extra sets of simulations run on our input data set of Day 1 and Day 2 with a varying amount of extra treatment rooms as an intervention. We, therefore, ran additional simulations of Scenario 3 above—for both Day 1 and Day 2—but now varying from two extra treatment rooms to six extra treatment rooms. The following scenarios will be denoted Scenario 3.2 (two extra treatment rooms), Scenario 3.4 (4 extra treatment rooms), and Scenario 3.6 (and extra treatment rooms). We see that in the emergency department, when the resources and demand are matched as on Day 1, most of the PIRs, at least up to 35%, do not yield a critical crowding level. On the high patient influx day, as in Day 2, the picture is quite different as the crowding is high within the whole range of PIRs.

To investigate deeper upon the notable effects of the introduced extra treatment rooms, we chose to make a more granular analysis by making a series of scenarios with different amounts of extra treatment rooms. Scenarios here are denoted 3.X as they all constitute a variation of Scenario 3 (please refer to Table 4 for the scenario design where this is also explained), which is the scenario in which we introduced the extra treatment room. Scenarios 3.2, 3.4, and 3.6 test the patient flow performance in the emergency department with two, four, and six extra treatment rooms. It is worth noting that Scenario 3.4 is the same as the original Scenario 3 as it has four extra treatment rooms and the same resource configuration.

#### 3.5.1. Patient Flows at an Average Patient Influx Day (Day 1)

**Figure 11:**
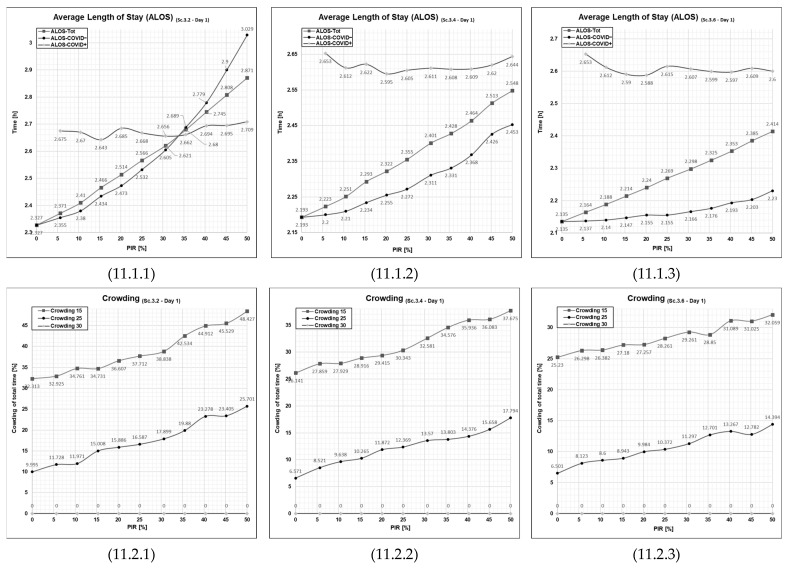
Graphical outputs from simulation runs illustrate increased PIR’s impact on both ALOS and crowding on Day 1 in Scenario 3.2, 3.4, and 3.6. Sc—scenario, Tot.—total patient population, Ord.—The patient agent group classified as COVID−, Con.—The patient agent group classified as COVID+, PIR—patient infection rate, h—number of hours. Cell references follows the following format (Figure No., Day; 1 = Day 1 or 2 = Day 2, PFPI; 1 = ALOS or 2 = Crowding). Numerical values are given for reference in Appendix A in Table A1, Table A2, Table A3 and Table A4.

At two extra treatment rooms, Scenario 3.2, Day 1 showed less favorable curves (Figure 11, cell 11.1.1), with ALOS for COVID− patients being a bit higher and climbing with increasing PIR at a steeper rate– as opposed to the flatter curves for scenario 3.4 (4 extra treatment rooms) (Figure 11, cell 11.1.2). In addition, crowding was at an overall higher level with two (Figure 11, cell 11.2.1) compared to four extra treatment rooms (Figure 11, cell 11.1.2). Scenario 3.6 (6 extra treatment rooms) showed even more favorable curves for both ALOS (Figure 11, cell 11.1.3) and crowding (Figure 11, cell 11.2.3) than for Scenario 3.2.

#### 3.5.2. Patient Flows at a High Patient Influx Day (Day 2)

**Figure 12:**
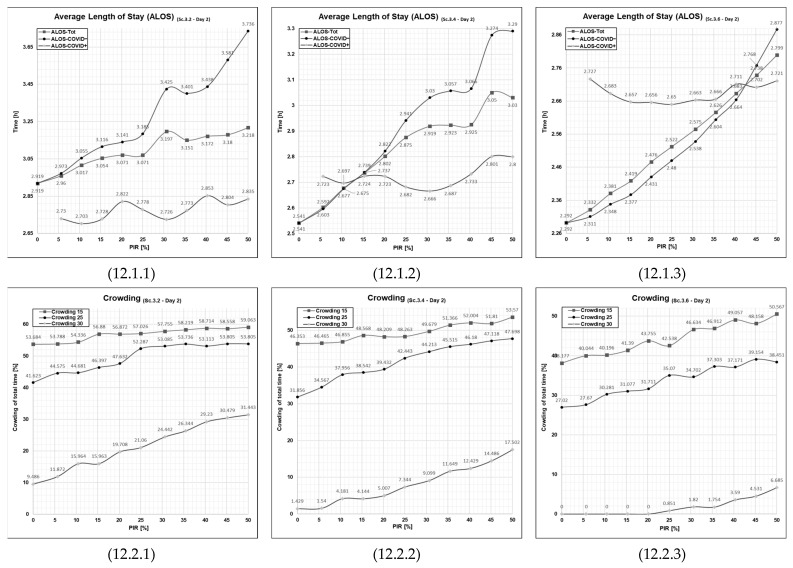
Graphical outputs from simulation run illustrating the impact of increased PIR on both ALOS and crowding in Day 2 in Scenario 3.2, 3.4 and 3.6. Sc—scenario, Tot.—total patient population, Ord.—the patient agent group classified as COVID−, Con.—the patient agent group classified as COVID+, PIR—patient infection rate, h—number of hours. Cell references follows the following format (Figure No., Day; 1 = Day 1 or 2 = Day 2, PFPI; 1 = ALOS or 2 = crowding). Numerical values are given for reference in Appendix A in Table A1, Table A2, Table A3 and Table A4.

At Scenario 3.2, Day 2, ALOS for both patient groups (Figure 12, cell 12.1.1), as well as crowding (Figure 12, cell 12.2.1), is at higher levels with only two compared to four extra treatment rooms. However, with six extra treatment rooms, ALOS for both patient groups (Figure 12, cell 12.1.1), as well as crowding (Figure 12, cell 12.2.1), are much improved.

The amount of extra treatment rooms one needs to introduce to obtain the desired beneficial effect on patient flow may thus depend on the level of the ED’s daily patient influx.

On the average patient influx day (Day 1) in our simulation, this effect was obtained when four extra treatment rooms were introduced. A further benefit was achieved with six extra treatment rooms. On the day of high patient influx (Day 2), six extra treatment rooms were needed to achieve a beneficial effect on ALOS and crowding comparable to that with four extra treatment rooms on a day of average patient influx (Day 1).

In addition, this set of curves indicates that when the patient influx is higher than the ED’s capacity to manage it, the patient flow performance indicators become elevated, tend to increase with increasing PIR and exhibit non-linear behavior. The degree of non-linear behavior diminishes when the amount of treatment rooms becomes adequate to handle the patient influx (the curves on the right-hand side of Figure 11 and Figure 12, cell 11.2.3 and 12.2.3).

### 3.6. Discussion of Collateral Impacts of the COVID-19 Pandemic

Previous studies have shown that the COVID-19 pandemic had a significant adverse collateral impact on the operation of emergency departments. For example, Cho et al., in the case of Korean emergency department patients, identified collateral impact on the emergency department patients in the form of increased in-house mortality during the pandemic [35]. In their conclusions, they recommend that further investigation should be performed to analyze the causes of the increased fatality. Furthermore, Cummins et al., in the case of an Irish emergency department, also found that the emergency department’s patient flow performance was adversely impacted by the COVID-19 pandemic [36]. In a similar respect, Cummins et al. also identified that there is an overall need for planning and preparedness to address such collateral impact from pandemics.

Furthermore, both research groups in the articles mentioned above pointed out a direct, second collateral impact on patient admissions to the emergency department. For example, Cummins et al. quantified a substantial influence attributed to the pandemic on a patients’ decision to seek and attend the emergency department. During the COVID-19 pandemic, despite being in need, patients were found to be less willing to seek and attend the emergency department, leading to a negative impact on fatality rates.

Although this study does not directly investigate such impacts on emergency department patient admission itself, the model does take into account a reduction in emergency department patient admissions. As pointed out in Section 2.5—‘Experimental Design and Simulation Model Scenarios’, the number of COVID− patients does decrease as the number of COVID+ patients increases. Hence, the model incorporated this collateral mentioned above effect caused by the COVID-19 pandemic.

Moreover, our findings show that interventions have a beneficial effect on the patient flow performance of COVID+ patients but at the expense of patient flow performance for COVID− patients. Specifically, the results show that COVID− patients in all scenarios will experience a higher average length of stay (ALOS), which is one of the many markers of patient dissatisfaction. In addition, as pointed out previously in the individual scenario discussions, the results showed that the patient flow performance measure ‘crowding’ in all scenarios significantly worsens with the increased patient infection rate (PIR). Please refer to subsections Section 3.1, Section 3.2, Section 3.3 and Section 3.4 of this article for exact quantities and research findings on the disparate scenario configurations and patient infection rates. Overall, higher crowding is associated with a higher strain on the emergency department resources leading to secondary collateral damages associated with high utilization of the emergency department, such as harder prioritization of the patient treatments, higher stress among the healthcare workers, longer time to diagnosis on patients, which may cause worsening in conditions, and many more [37].

Hence, on the results and findings in the present article and because of the explanation given above, we believe our computerized simulation model and this study is relevant and contributes to each of the above-listed collateral impacts on patient flow in the emergency department. Thus, we believe when applied on-site, along with findings such as those above-presented findings, the computerized simulation model can be helpful for practitioners to plan what interventions to introduce to the emergency department in order to identify, address, and reduce such adverse collateral impact within their emergency departments.

### 3.7. Limitations

Due to practicality, this study does not consider every possible patient flow performance indicator. Instead, it delimits its focus on the select subset of the performance indicators. Performance indicators that are considered to be of interest may differ among organizations; hence these particular patient flow performance indicators may be viewed as irrelevant or not viewed to be fitting for other emergency departments.

This simulation modeling study does not take into account the cost of each of the interventions. Even though we see a far better improvement in introducing the extra treatment rooms for all rates of patient infection rates, the model does not take into account the increased cost of doing so. It is likely that this trend would be amplified with an even higher amount of extra treatment rooms. At the final stage of analysis, parameters should be taken into consideration.

### 3.8. Suggestions for Further Research Work

A model of the type used in this study may be used in conjunction with a predictive model of patient inflow, such as the model described in the workings of Skinner et al. [38]. Assuming that the given health authorities track the given PIR of the hospital regions, that result could be combined with the model used in this present study and then lead to a tailored response to that particular patient infection rate of that given day. An example could be preemptively adjusting the resources, e.g., the number of extra treatment rooms or the number of spots used in the waiting zone to the necessary level so that the emergency department during this day, e.g., never crosses the crowding 30 level.

The next logical step could be to combine the model with any predictive model that estimates the number of patients arriving at the emergency department, such as the workings of Phan et al. [39]. This article has, through the patient flow performance indicators, highlighting the disparate burden the emergency department experiences during a day of average patient influx and a day of high patient influx. If there was to be found a reliable model to predict a probable range of patient influx for any given day, the exemplified above-mentioned strategy could be used for the same balancing purpose.

## 4. Conclusions

The COVID-19 pandemic put the global healthcare system in a new and uncertain situation. In many ways, it revealed how fragile it was for such an unprecedented healthcare crisis and that it could drastically affect the systems even in these modern times. Now, gathering knowledge from the situation is more important than ever to learn from what has happened.

To investigate the effect of pandemic conditions on the emergency healthcare, the present study endeavored to answer the twofold research question posed in the introductory section of this manuscript. Firstly, investigating the impact of increased patient infection rate on the emergency department patient flow parameters, and secondly, examining the likely impact of increased patient infection rate on patient flow parameters in an emergency department. The results of this study have shown that for every configuration of the emergency department, an increment in all instances will lead to an incremental worsening of the patient flow metrics average length of stay and crowding within the emergency department. Depending on the scenario, some configurations have less sensitivity to patient infection rate while others have a larger sensitivity to the increase in patient infection rate. The highest sensitivity is found in the scenario that only includes a waiting zone to the emergency department, Scenario 2, which counterintuitively has an even higher sensitivity than the base case, which does not have any of the pandemic interventions implemented this holds both for the low and high influx days.

On the other hand, the lowest sensitivity to patient infection rate is found when only introducing the extra treatment rooms, Scenario 3, to the emergency department patient flow. This is also interestingly contrary to what one would expect, as one would find it more likely that the emergency department would run the best with both interventions implemented.

Although the lower patient infection rate sensitivity is found to be, Scenario 3, where only extra treatment rooms are introduced, it remains a question of finding a balance between patient cross-infection risk and patient flow performance, which strictly is not within the scope of this study to find the balance as this necessitates a holistic approach with departmental leaders knowledgeable and decision competence on the matter.

It was found that, particularly in Scenario 4, with the active waiting zone and extra treatment room interventions, it was not the scenario with the best patient infection rate sensitivity. However, the disparity was far less pronounced on a low influx day. Having this knowledge that this certain configuration is less sensitive towards an increase in patient infection rate when influx is low may inform emergency department leaders that different strategies have different outcomes depending on the particularities of the patient influx. If a model, such as the one utilized, was to be combined with a big-data model predicting patient influx for a certain date, it could lead to proliferating findings.

## Figures and Tables

**Table 1:** Tabulated summary resources aimed to reduce the risk of intradepartmental cross-infection.

Abbreviation	Resource Description
**PT**	Pre-Triage: The pre-triage is an introduced physical location outside of the emergency department. Admitted patients enter here for the healthcare workers to assess whether the incoming patient should be regarded as infected by COVID-19 (COVID+) or not infected by COVID-19 (COVID−). Depending on the status, the patient will, in the case of COVID−, be directed the regular route via the emergency department reception or, in the case of COVID+, be fast-tracked directly to an available treatment room for proper isolation and treatment of the emergent condition the patient suffers.
**E.TR.**	Extra Treatment Rooms: Because of the COVID-19 pandemic situation, extra treatment rooms were introduced to be available for the emergency department. These additional treatment rooms were introduced to compensate for the higher occupancy of patients in the emergency department at the onset of the pandemic. In the case of the emergency department, they introduced four extra-treatment rooms.
**WZ**	A designated area was allocated for the precise purpose of enabling the fast-tracking of the patients classified as COVID+. In the instance where a patient who is COVID− has completed their treatment and requires either restitution or non-intensive follow-up, the treatment room may be deemed unnecessary for their further stay in the emergency department. Suppose no further treatment rooms are available for incoming COVID+ patients entering the emergency department. In that case, the COVID− patient may vacate the treatment room to allow for an incoming COVID+ patient to expeditiously receive proper isolation, thus minimizing cross-infection among other patients and healthcare workers within the emergency department.

**Table 2:** Overview of the selected patient flow performance indicators used in this study.

Abbreviation	Patient Flow Performance Indicators (PFPI) and Description	Unit
**TTT**	Time to Treatment: This performance indicator calculates the patient population’s average time within the emergency department before the treatment starts in their assigned treatment room. The value is in this simulation study given on a per-patient basis, i.e., the number of hours of waiting per patient. A lower value is better, all else equal, compared to a higher value, as less waiting means that each patient faster receives health care for the condition they got admitted for. This patient performance indicator is reported for the aggregate total group of patient agents (Tot.), the patients classified with the COVID+ status, and the patients classified with the COVID− status.	Hours per patient [h/pt]
**ALOS**	The average length of stay: The performance indicator is calculated, showing the total amount of time spent in the emergency department on a per-patient basis. It tracks the time taken from entering the emergency department, the point of admission, until the patient has been discharged from the emergency department. The performance indicator is measured as an average for all the patient agents, meaning the value gives the average hours of time each patient spent in the emergency department. Similar to the previous patient flow performance indicator, TTT, this performance indicator is also reported for the aggregate total group of patient agents (Tot.), the patients classified with the COVID+ status, and the patients classified with the COVID− status.	Hours per patient [h/pt]
**Crowding**	The patient flow performance indicator called ‘crowding’ calculates for each and every moment how many patients are present in the emergency department. This patient flow performance indicator is, including its naming, from the case emergency department’s patient flow improvements project. In the implementation of this metric, we show how much of the total simulated time the crowding has been beyond three different thresholds of interest as defined by the case emergency department in their internal working emphasis on the improvement of patient flow conditions and response for over-crowding problematics. The three threshold levels are set at 15, 25, and 30 patients in the emergency department.	%
**Peak Crowding**	Peak crowding, as its name suggests, is concerned about the peak of crowding, the previous performance indicator. The given number (#) tells how many patients were residing at the emergency department at the point in time when the number was at its maximum that day. In addition, the metric also tracks the time during the day the peak occurs. Thus, the first portion states the number of patients, while the second portion states how much the clock was at that given point.	# & Time
**Time Start Use/Time in Use**	This patient flow performance indicator states the time different resources of particular interest start being used during the day. In this performance measure implementation, it has been chosen to track when the extra treatment rooms (E.TR.), triage (Tri.), and waiting zone (WZ) are used. Secondly, the performance indicator calculates how much the given resource is used in proportion to the total elapsed time of that time, e.g., a reading in the Tri.-column at ‘11:00′ means that the triage that day was not used until 11:00. The second value shows the percentage amount of that day this resource has been used at least by one patient.	Time
**Time Full**	This performance measure, as the name suggests, keeps track of when particular resources of interest, the extra treatment rooms (E.TR.), triage (Tri.), and waiting zone (WZ), are full, meaning they cannot further take any more patients. Similar to the previous performance indicator, this also has dual tracking. Firstly, the time when the given resource is full is given next, a percentage of how long the elapsed time the resource has been full.	Time & %
**Time in WR**	This performance measure calculates how long patients on average, spent in the emergency department waiting room. The indicator is calculated as an average across all the patients.	h
**Times TR blocked** **for COVID+.**	This performance indicator tracks the total number of times (#) a patient agent classified as COVID+ is blocked from entering the treatment room after pre-triage. This is a critical point for the emergency department as it is heavily loaded with the patients so much that they cannot immediately fast-track a patient to an isolated room.	#
**Times TR** **Assigned**	This performance measure tracks the number of times (#) a treatment room (TR) has been assigned to a patient. This refer to the term ‘seize’ used in simulation modeling discipline; how many times a certain resource is utilized for the purpose treatment of a patient. Each time a patient is assigned to a treatment room is resource-consuming, especially considering the sanitization of the rooms.	#

**Table 3:** Overview of the different classes of patient agents in the computerized simulation model.

Abbreviations	Model Inputs Description
**Tot.**	Total patient population: This denotes both the patient groups who are classified as COVID+ and COVID−. This category was made to show the combined effects of the total patient groups.
**COVID−**	This is the population of patient agents that in the pre-triage is deemed to not be infected by COVID-19. These patients will, after the pre-triage control, progress to the emergency department in the usual manner.
**COVID+**	This points to the portion of the patient agents that in the pre-triage is found to be infected by COVID-19. As these patient agents pose an increased risk associated with the infection status, the patient agents will be fast-tracked to a treatment room.
**PIR**	Patient infection rate: The variable determining the proportion of how many COVID+ patients will be inserted in the different simulation runs. In the present study, the PIR is varied between 0 and 50%, with a 5 percentage points increment between each run.

**Table 4:** Table listing and summarizing the scenario series performed in this study detailing purpose and showing their configurations.

Sc. No.	Explanation	Model and Resource Configuration		
		PIR [%]	WZ	E.TR.
Sc. 1	Base case—no added resources: Scenario simulating situation during pandemic operation. However, none of the extra resources are introduced. What are introduced in this series of scenarios are only the new policies, i.e., channeling and expediting the COVID+ patients according to the infection control policies. Thus, the E.TR. and WZ are not in use for this scenario.	0 → 50		
Sc. 2	Adding the waiting zone (WZ): A simulation series runs with a single parameter variation. Peri-pandemic operation with differing proportions of patient infection rates: 0–50% with 5 percentage point increments between each simulation run. This series of scenario runs has the waiting zone (WZ) enabled for utilization when the circumstances are as described in Section 2.4—‘Case Emergency Department, Interventions, and Resources’ and listed in Table 1.	0 → 50	✓	
Sc. 3	Adding extra treatment rooms: A series of simulation runs with single parameter variation. Peri-pandemic operation with differing proportions of patient infection rates: 0–50% with 5 percentage point increments between each simulation run. This series of scenario runs has the extra treatment rooms (E.TR.) enabled for utilization.	0 → 50		✓
Sc. 3.2 Sc. 3.4 Sc. 3.6	A sub-group of three simulation series of simulation runs: measure the differential impact of the increased number of E.TR.; In Sc. 3.2, the number of E.TR is two; in Sc. 3.4, the number of E.TR. is four; lastly, in Sc. 3.6 the number of E.TR. is six.	0 → 50		✓
Sc. 4	Adding waiting zone and extra treatment rooms: series of simulation runs with single parameter variation. Peri-pandemic operation with differing proportions of patient infection rates: 0–50% with 5 percentage point increments between each simulation run. This series of scenario runs includes both the WZ and the E.TR. enabled for utilization.	0 → 50	✓	✓

Sc. No.—scenario number, PIR—patient infection rate, E.TR.—extra treatment rooms, WZ—waiting zone, ✓ —indicates inclusion of resource.

## Data Availability

The numerical data used in this study are not publicly available and thus are not for distribution.

## Abbreviations

ABM—agent-based modeling, DES—discrete event simulation, ED—emergency department, PF—patient flow, SUS—Stavanger Universitetssykehus (The University Hospital of Stavanger), WZ—waiting zone, TR—treatment room, Tri.—triage, PT—pre-triage, E.TR.—extra treatment room, WR—waiting room, PF—patient flow, PIR—patient infection rate, TTT—time to treatment, ALOS—average length of stay.

## Appendix A

The tabulated numerical output retrieved from the simulation output is disclosed in the appendix. The data in the following tables, Table A1, Table A2, Table A3 and Table A4, is the underlying data for the results presented in the present manuscript, where important values among them are referenced in the main text for precise reference. The data is given here for the purpose of research transparency.

**Table A1:** Curated output from Sc. 1, parameter variation test 5% increments on PIR [0–50%].

Day 1	Time to Treatment [h/pt]			ALOS [h/pt]			Crowding [%]			Peak Crowding ED [#] (Time of Peak [When])	Time Start Use [When] (Time in Use [%])			Time Full [When] (Time Full [%])			Time in WR (h/pt)	Times TR Blocked for cont. (#)	Times TR Seized [#] (Times WZ Seized [#])
PCR	Tot.	Ord.	Con.	Tot.	Ord.	Con.	>15	>25	>30	ED	E. TR	Tri.	WZ	E. TR	Tri.	WZ			
**0%**	0.674	0.674	null	2.699	2.699	null	42.379 (11:00)	20.182 (13:30)	0.0 (null)	26 (14:45)	null (0.0)	11:00 (52.876)	null (0.0)	null (0.0)	14:19 (4.697)	null (0.0)	0.034	0	124 (0)
**5%**	0.716	0.76	0.114	2.775	2.785	2.645	44.537 (11:00)	20.759 (12:15)	0.0 (null)	26 (14:45)	null (0.0)	11:00 (55.295)	null (0.0)	null (0.0)	14:18 (5.162)	null (0.0)	0.035	4	124 (0)
**10%**	0.776	0.858	0.169	2.861	2.883	2.698	46.383 (11:00)	24.992 (12:15)	0.0 (null)	27 (14:45)	null (0.0)	11:00 (56.572)	null (0.0)	null (0.0)	14:18 (6.327)	null (0.0)	0.037	10	124 (0)
**15%**	0.824	0.916	0.141	2.909	2.941	2.67	46.907 (11:00)	25.776 (13:30)	0.0 (null)	27 (14:45)	null (0.0)	11:00 (55.911)	null (0.0)	null (0.0)	14:18 (9.719)	null (0.0)	0.051	10	124 (0)
**20%**	0.896	1.038	0.178	3.004	3.063	2.708	47.207 (11:00)	27.954 (13:30)	0.0 (null)	29 (14:45)	null (0.0)	11:00 (58.642)	null (0.0)	null (0.0)	14:18 (10.745)	null (0.0)	0.069	13	124 (0)
**25%**	0.984	1.257	0.22	3.141	3.281	2.75	57.214 (11:00)	33.357 (12:00)	0.0 (null)	30 (15:30)	null (0.0)	11:00 (61.842)	null (0.0)	null (0.0)	13:33 (17.229)	null (0.0)	0.122	22	124 (0)
**30%**	0.999	1.407	0.255	3.202	3.432	2.784	54.681 (11:00)	33.621 (12:15)	0.0 (null)	30 (14:45)	null (0.0)	11:00 (64.57)	null (0.0)	null (0.0)	14:18 (14.185)	null (0.0)	0.099	28	124 (0)
**35%**	1.057	1.591	0.275	3.287	3.617	2.803	56.765 (11:00)	35.211 (13:30)	1.749 (15:45)	31 (15:45)	null (0.0)	11:00 (65.214)	null (0.0)	null (0.0)	14:18 (19.983)	null (0.0)	0.109	33	124 (0)
**40%**	1.024	1.587	0.265	3.264	3.612	2.794	57.004 (11:00)	38.161 (12:15)	0.688 (15:30)	31 (15:30)	null (0.0)	11:00 (66.703)	null (0.0)	null (0.0)	14:18 (20.97)	null (0.0)	0.233	34	121 (0)
**45%**	1.117	1.821	0.288	3.373	3.846	2.817	60.003 (11:00)	38.375 (12:00)	1.601 (15:45)	32 (15:45)	null (0.0)	11:00 (65.291)	null (0.0)	null (0.0)	14:03 (18.773)	null (0.0)	0.182	35	124 (0)
**50%**	1.074	2.076	0.294	3.383	4.102	2.823	61.473 (11:00)	42.044 (12:00)	1.439 (14:45)	31 (14:45)	null (0.0)	11:00 (68.781)	null (0.0)	null (0.0)	14:18 (20.106)	null (0.0)	0.265	43	124 (0)
**Day 2**																			
**0%**	1.096	1.096	null	3.121	3.121	null	58.518 (11:45)	51.829 (12:30)	26.606 (15:00)	39 (17:15)	null (0.0)	11:33 (63.199)	null (0.0)	null (0.0)	12:48 (39.28)	null (0.0)	0.259	0	138 (0)
**5%**	1.176	1.281	0.113	3.247	3.306	2.643	59.415 (11:45)	52.191 (12:30)	30.51 (15:00)	40 (18:15)	null (0.0)	11:33 (63.194)	null (0.0)	null (0.0)	12:48 (41.272)	null (0.0)	0.304	10	135 (0)
**10%**	1.085	1.193	0.389	3.178	3.218	2.919	60.196 (11:45)	52.337 (12:30)	30.127 (15:00)	41 (17:00)	null (0.0)	11:33 (64.909)	null (0.0)	null (0.0)	13:03 (40.822)	null (0.0)	0.256	14	134 (0)
**15%**	1.151	1.391	0.2	3.278	3.417	2.729	61.225 (11:45)	52.543 (12:15)	32.795 (15:00)	41 (16:45)	null (0.0)	11:33 (63.284)	null (0.0)	null (0.0)	12:48 (40.797)	null (0.0)	0.221	17	135 (0)
**20%**	1.124	1.372	0.255	3.261	3.397	2.785	60.883 (11:45)	52.229 (12:30)	32.268 (15:00)	43 (19:30)	null (0.0)	11:33 (64.901)	null (0.0)	null (0.0)	13:18 (40.597)	null (0.0)	0.261	18	132 (0)
**25%**	1.016	1.279	0.341	3.182	3.304	2.87	61.045 (11:45)	52.692 (12:30)	36.474 (15:00)	44 (17:30)	null (0.0)	11:33 (64.99)	null (0.0)	null (0.0)	13:18 (38.655)	null (0.0)	0.297	28	127 (0)
**30%**	1.27	1.914	0.246	3.49	3.94	2.775	64.286 (11:45)	53.156 (12:15)	37.457 (15:00)	45 (17:30)	null (0.0)	11:33 (65.432)	null (0.0)	null (0.0)	12:48 (45.128)	null (0.0)	0.334	32	127 (0)
**35%**	1.001	1.663	0.225	3.258	3.689	2.754	63.777 (11:45)	53.146 (12:15)	37.457 (15:00)	45 (17:30)	null (0.0)	11:33 (66.343)	null (0.0)	null (0.0)	12:48 (45.749)	null (0.0)	0.391	39	126 (0)
**40%**	1.099	2.169	0.313	3.414	4.193	2.842	66.159 (11:30)	54.667 (12:15)	37.933 (14:45)	47 (17:30)	null (0.0)	11:33 (66.982)	null (0.0)	null (0.0)	13:33 (42.714)	null (0.0)	0.544	46	124 (0)
**45%**	1.161	1.977	0.33	3.436	4.002	2.859	65.816 (11:45)	55.169 (12:15)	37.457 (15:00)	46 (17:30)	null (0.0)	11:33 (67.203)	null (0.0)	null (0.0)	13:48 (41.513)	null (0.0)	0.372	37	127 (0)
**50%**	0.915	2.179	0.509	3.322	4.204	3.038	66.037 (11:45)	56.143 (12:15)	38.057 (14:45)	50 (20:45)	null (0.0)	11:33 (68.342)	null (0.0)	null (0.0)	13:33 (43.078)	null (0.0)	0.039	59	124 (0)

PCR—Patient contamination rate, Tot.—Total patient population, Ord.—Ordinary patients without suspicion of being pathogen contagious, Con.—Patients categorized as being with suspicion pathogen contagion, ED—Emergency department, E.Tr.—Extra treatment rooms, Tri.—Triage, WZ—Waiting zone, WR—Waiting room.

**Table A2:** Curated output from Sc. 2, parameter variation test 5% increments on PIR [0–50%].

Day 1	Time to Treatment [h/pt]			ALOS [h/pt]			Crowding [%]			Peak Crowding ED [#] (Time of Peak [When])	Time Start Use [When] (Time in Use [%])			Time Full [When] (Time Full [%])			Time in WR (h/pt)	Times TR Blocked for cont. (#)	Times TR Seized [#] (Times WZ Seized [#])
PCR	Tot.	Ord.	Con.	Tot.	Ord.	Con.	>15	>25	>30	ED	E. TR	Tri.	WZ	E. TR	Tri.	WZ			
**0%**	0.674	0.674	null	2.699	2.699	null	42.379 (11:00)	20.182 (13:30)	0.0 (null)	26 (14:45)	null (0.0)	11:00 (52.876)	null (0.0)	null (0.0)	14:19 (4.697)	null (0.0)	0.034	0	124 (0)
**5%**	0.704	0.734	0.028	2.75	2.759	2.559	44.838 (11:00)	21.362 (12:15)	0.0 (null)	26 (14:45)	null (0.0)	11:00 (53.752)	17:30 (0.0)	null (0.0)	14:18 (5.162)	null (0.0)	0.035	0	125 (1)
**10%**	0.766	0.842	0.028	2.839	2.868	2.558	45.567 (11:00)	24.282 (13:30)	0.0 (null)	27 (15:30)	null (0.0)	11:00 (56.789)	13:45 (0.0)	null (0.0)	14:18 (5.766)	null (0.0)	0.04	0	132 (8)
**15%**	0.771	0.847	0.028	2.844	2.873	2.557	45.306 (11:00)	24.141 (13:30)	0.0 (null)	27 (15:30)	null (0.0)	11:00 (56.803)	13:45 (0.0)	null (0.0)	14:18 (5.766)	null (0.0)	0.04	0	131 (7)
**20%**	0.979	1.181	0.03	3.094	3.208	2.56	53.5 (11:00)	32.911 (11:30)	0.0 (null)	30 (15:45)	null (0.0)	11:00 (60.379)	11:00 (0.0)	null (0.0)	14:18 (12.496)	null (0.0)	0.084	2	140 (16)
**25%**	0.97	1.2	0.031	3.095	3.226	2.561	50.377 (11:00)	30.928 (12:15)	0.0 (null)	30 (14:45)	null (0.0)	11:00 (61.067)	11:00 (0.0)	null (0.0)	14:18 (11.23)	null (0.0)	0.089	3	140 (16)
**30%**	1.057	1.487	0.045	3.234	3.515	2.574	57.362 (11:00)	35.921 (12:00)	3.04 (14:30)	34 (14:45)	null (0.0)	11:00 (61.926)	11:00 (2.341)	null (0.0)	14:35 (15.781)	14:15 (0.0)	0.115	8	145 (21)
**35%**	1.16	1.803	0.057	3.373	3.832	2.586	58.932 (11:00)	39.084 (12:15)	0.949 (14:45)	32 (14:45)	null (0.0)	11:00 (62.505)	11:00 (0.0)	null (0.0)	14:20 (18.893)	null (0.0)	0.159	12	147 (23)
**40%**	1.232	2.166	0.079	3.486	4.196	2.609	64.307 (11:00)	42.508 (11:30)	1.669 (14:45)	32 (14:45)	null (0.0)	11:00 (64.334)	11:00 (0.0)	null (0.0)	14:05 (20.459)	null (0.0)	0.226	15	154 (30)
**45%**	1.179	2.199	0.082	3.451	4.231	2.611	63.18 (11:00)	45.553 (12:00)	2.433 (14:30)	32 (14:45)	null (0.0)	11:00 (66.505)	11:00 (0.0)	null (0.0)	13:48 (25.838)	null (0.0)	0.27	17	154 (33)
**50%**	0.989	2.049	0.168	3.301	4.082	2.697	64.686 (11:00)	45.038 (11:30)	1.668 (14:30)	33 (14:45)	null (0.0)	11:00 (69.976)	11:00 (0.0)	null (0.0)	13:50 (27.207)	null (0.0)	0.343	28	156 (33)
**Day 2**																			
**0%**	1.096	1.096	null	3.121	3.121	null	58.518 (11:45)	51.829 (12:30)	26.606 (15:00)	39 (17:15)	null (0.0)	11:33 (63.199)	null (0.0)	null (0.0)	12:48 (39.28)	null (0.0)	0.259	0	138 (0)
**5%**	1.238	1.356	0.028	3.308	3.382	2.558	59.819 (11:45)	52.629 (12:30)	29.551 (15:00)	41 (17:30)	null (0.0)	11:33 (63.194)	11:45 (0.0)	null (0.0)	12:48 (40.724)	null (0.0)	0.352	0	144 (8)
**10%**	1.076	1.201	0.03	3.156	3.227	2.558	59.276 (11:45)	52.726 (12:30)	29.776 (15:00)	41 (17:00)	null (0.0)	11:33 (64.964)	12:45 (0.0)	null (0.0)	13:03 (42.865)	null (0.0)	0.21	1	146 (10)
**15%**	1.216	1.437	0.028	3.322	3.464	2.556	59.612 (11:45)	53.622 (12:30)	31.446 (15:00)	42 (17:30)	null (0.0)	11:33 (65.97)	13:00 (0.0)	null (0.0)	12:48 (42.227)	null (0.0)	0.327	0	148 (13)
**20%**	1.212	1.604	0.039	3.367	3.633	2.568	61.392 (11:45)	53.888 (12:30)	35.687 (15:00)	46 (17:30)	null (0.0)	11:33 (64.683)	11:45 (0.0)	null (0.0)	13:03 (39.153)	null (0.0)	0.259	8	158 (25)
**25%**	1.205	1.59	0.063	3.36	3.618	2.593	60.794 (11:45)	54.584 (12:15)	35.962 (15:00)	45 (17:30)	null (0.0)	11:33 (66.281)	11:45 (0.0)	null (0.0)	13:04 (41.051)	null (0.0)	0.279	7	152 (23)
**30%**	1.3	1.959	0.064	3.504	3.99	2.592	64.576 (11:45)	54.954 (12:30)	36.761 (15:00)	44 (17:00)	null (0.0)	11:33 (66.798)	11:45 (0.0)	null (0.0)	13:33 (41.286)	null (0.0)	0.441	12	160 (32)
**35%**	1.338	2.262	0.074	3.578	4.291	2.604	63.709 (11:45)	54.547 (12:30)	38.723 (13:45)	45 (16:45)	null (0.0)	11:33 (68.524)	11:45 (2.444)	null (0.0)	13:50 (40.626)	13:15 (0.0)	0.45	16	158 (28)
**40%**	1.338	2.599	0.122	3.623	4.631	2.651	65.434 (11:45)	55.725 (12:15)	37.457 (15:00)	50 (17:30)	null (0.0)	11:33 (68.349)	12:00 (2.482)	null (0.0)	13:03 (43.676)	15:31 (0.0)	0.424	16	162 (37)
**45%**	1.27	2.657	0.192	3.582	4.689	2.721	67.118 (11:45)	55.725 (12:15)	37.457 (15:00)	50 (19:30)	null (0.0)	11:33 (70.772)	12:00 (12.209)	null (0.0)	13:03 (41.651)	15:30 (0.0)	0.32	27	155 (30)
**50%**	0.929	2.529	0.106	3.291	4.565	2.636	66.839 (11:30)	56.3 (12:15)	39.062 (13:45)	48 (18:15)	null (0.0)	11:33 (67.85)	12:00 (0.372)	null (0.0)	13:33 (43.083)	13:45 (0.0)	0.164	25	162 (40)

PCR—Patient contamination rate, Tot.—Total patient population, Ord.—Ordinary patients without suspicion of being pathogen contagious, Con.—Patients categorized as being with suspicion pathogen contagion, ED—Emergency department, E.Tr.—Extra treatment rooms, Tri.—Triage, WZ—Waiting zone, WR—Waiting room.

**Table A3:** Curated output from Sc. 3, parameter variation test 5% increments on PIR [0–50%].

Day 1	Time to Treatment [h/pt]			ALOS [h/pt]			Crowding [%]			Peak Crowding ED [#] (Time of Peak [When])	Time Start Use [When] (Time in Use [%])			Time Full [When] (Time Full [%])			Time in WR (h/pt)	Times TR Blocked for cont. (#)	Times TR Seized [#] (Times WZ Seized [#])
PCR	Tot.	Ord.	Con.	Tot.	Ord.	Con.	>15	>25	>30	ED	E. TR	Tri.	WZ	E. TR	Tri.	WZ			
**0%**	0.165	0.165	null	2.193	2.193	null	26.212 (11:00)	6.598 (14:45)	0.0 (null)	23 (15:15)	11:00 (44.386)	12:03 (15.609)	null (0.0)	11:00 (28.482)	null (0.0)	null (44.386)	0.028	0	124 (0)
**5%**	0.175	0.181	0.028	2.223	2.209	2.56	26.755 (11:00)	7.811 (12:15)	0.0 (null)	23 (15:15)	11:00 (44.406)	12:03 (18.12)	null (0.0)	11:00 (28.507)	null (0.0)	null (44.406)	0.028	0	124 (0)
**10%**	0.181	0.186	0.139	2.26	2.213	2.669	27.939 (11:00)	8.427 (12:15)	0.0 (null)	23 (14:45)	11:00 (44.392)	11:03 (18.87)	null (0.0)	11:00 (28.462)	null (0.0)	null (44.392)	0.028	6	124 (0)
**15%**	0.196	0.219	0.086	2.309	2.246	2.617	29.535 (11:00)	9.68 (14:30)	0.0 (null)	25 (15:30)	11:00 (47.51)	12:03 (18.66)	null (0.0)	11:00 (29.757)	null (0.0)	null (47.51)	0.028	8	124 (0)
**20%**	0.203	0.231	0.054	2.311	2.258	2.585	29.42 (11:00)	10.91 (12:15)	0.0 (null)	24 (14:45)	11:00 (46.275)	12:03 (21.252)	null (0.0)	11:00 (28.675)	null (0.0)	null (46.275)	0.028	3	124 (0)
**25%**	0.202	0.232	0.067	2.323	2.259	2.598	28.564 (11:00)	10.584 (12:15)	0.0 (null)	23 (14:15)	11:00 (49.405)	12:03 (21.731)	null (0.0)	11:00 (28.661)	null (0.0)	null (49.405)	0.028	6	124 (0)
**30%**	0.211	0.243	0.128	2.377	2.27	2.658	30.745 (11:00)	12.229 (13:45)	0.0 (null)	23 (14:45)	11:00 (46.351)	11:00 (21.089)	null (0.0)	11:00 (28.668)	null (0.0)	null (46.351)	0.028	8	124 (0)
**35%**	0.244	0.324	0.106	2.455	2.35	2.637	35.734 (11:00)	14.392 (12:00)	0.0 (null)	25 (14:45)	11:00 (47.67)	11:33 (24.815)	null (0.0)	11:00 (32.844)	null (0.0)	null (47.67)	0.028	13	124 (0)
**40%**	0.243	0.34	0.097	2.47	2.367	2.627	34.264 (11:00)	15.922 (12:00)	0.0 (null)	25 (15:45)	11:00 (50.027)	12:03 (23.69)	null (0.0)	11:00 (34.072)	null (0.0)	null (50.027)	0.028	14	124 (0)
**45%**	0.274	0.414	0.119	2.54	2.442	2.649	35.515 (11:00)	17.864 (12:15)	0.0 (null)	26 (14:45)	11:00 (50.657)	12:03 (24.881)	null (0.0)	11:00 (33.71)	null (0.0)	null (50.657)	0.028	18	124 (0)
**50%**	0.265	0.381	0.151	2.546	2.409	2.68	34.927 (11:00)	17.119 (13:45)	0.0 (null)	26 (15:45)	11:00 (50.989)	11:03 (24.835)	null (0.0)	11:00 (33.548)	null (0.0)	null (50.989)	0.028	22	124 (0)
**Day 2**																			
**0%**	0.514	0.514	null	2.541	2.541	null	46.305 (11:45)	31.866 (12:30)	1.355 (17:15)	31 (17:15)	11:33 (50.352)	12:18 (41.288)	null (0.0)	11:48 (46.744)	15:18 (3.031)	null (50.352)	0.038	0	146 (0)
**5%**	0.589	0.626	0.114	2.652	2.652	2.644	47.581 (11:45)	35.333 (12:30)	3.535 (15:30)	34 (17:30)	11:33 (57.59)	12:18 (43.765)	null (0.0)	11:48 (47.055)	15:18 (6.392)	null (57.59)	0.053	7	146 (0)
**10%**	0.647	0.729	0.186	2.75	2.756	2.718	47.755 (11:45)	38.239 (12:30)	5.356 (15:30)	34 (17:30)	11:33 (57.577)	12:18 (45.799)	null (0.0)	11:48 (48.158)	15:18 (8.703)	null (57.577)	0.06	11	146 (0)
**15%**	0.655	0.755	0.168	2.768	2.782	2.697	47.844 (11:45)	41.006 (12:15)	4.133 (15:15)	35 (17:00)	11:33 (52.309)	12:18 (43.965)	null (0.0)	11:48 (46.861)	15:03 (9.531)	null (52.309)	0.07	14	146 (0)
**20%**	0.692	0.824	0.201	2.826	2.851	2.731	47.813 (11:45)	40.281 (12:30)	5.037 (15:15)	35 (17:15)	11:33 (59.423)	12:18 (45.878)	null (0.0)	11:48 (48.204)	15:18 (9.743)	null (59.423)	0.067	17	146 (0)
**25%**	0.692	0.824	0.201	2.826	2.851	2.731	47.813 (11:45)	40.281 (12:30)	5.037 (15:15)	35 (17:15)	11:33 (59.423)	12:18 (45.878)	null (0.0)	11:48 (48.204)	15:18 (9.743)	null (59.423)	0.067	17	146 (0)
**30%**	0.738	0.991	0.185	2.923	3.019	2.715	49.347 (11:30)	43.576 (12:15)	6.922 (15:15)	34 (17:00)	11:33 (73.999)	12:18 (45.724)	null (0.0)	11:48 (48.263)	15:18 (14.42)	null (73.999)	0.088	24	146 (0)
**35%**	0.727	1.091	0.221	2.965	3.118	2.751	53.815 (11:30)	45.377 (12:15)	13.294 (15:15)	36 (17:00)	11:33 (74.99)	12:18 (47.386)	null (0.0)	11:48 (56.18)	15:18 (18.36)	null (74.99)	0.121	35	146 (0)
**40%**	0.857	1.331	0.179	3.091	3.359	2.71	54.879 (11:30)	46.072 (12:15)	14.001 (15:15)	36 (17:30)	11:33 (73.043)	12:18 (48.971)	null (0.0)	11:48 (55.009)	15:04 (18.712)	null (73.043)	0.194	33	146 (0)
**45%**	0.84	1.393	0.234	3.107	3.42	2.764	53.371 (11:30)	47.933 (12:15)	19.29 (15:15)	39 (18:15)	11:33 (73.035)	12:18 (49.939)	null (0.0)	11:46 (51.908)	15:48 (19.507)	null (73.035)	0.227	38	146 (0)
**50%**	0.827	1.488	0.318	3.139	3.517	2.847	54.228 (11:30)	48.574 (12:15)	15.906 (15:15)	38 (17:30)	11:33 (76.708)	12:18 (50.2)	null (0.0)	11:48 (53.962)	15:48 (18.469)	null (76.708)	0.172	47	146 (0)

PCR—Patient contamination rate, Tot.—Total patient population, Ord.—Ordinary patients without suspicion of being pathogen contagious, Con.—Patients categorized as being with suspicion pathogen contagion, ED—Emergency department, E.Tr.—Extra treatment rooms, Tri.—Triage, WZ—Waiting zone, WR—Waiting room.

**Table A4:** Curated output from Sc. 4, parameter variation test 5% increments on PIR [0–50%].

Day 1	Time to Treatment [h/pt]			ALOS [h/pt]			Crowding [%]			Peak Crowding ED [#] (Time of Peak [When])	Time Start Use [When] (Time in Use [%])			Time Full [When] (Time Full [%])			Time in WR (h/pt)	Times TR Blocked for cont. (#)	Times TR Seized [#] (Times WZ Seized [#])
PCR	Tot.	Ord.	Con.	Tot.	Ord.	Con.	>15	>25	>30	ED	E. TR	Tri.	WZ	E. TR	Tri.	WZ			
**0%**	0.165	0.165	null	2.193	2.193	null	26.212 (11:00)	6.598 (14:45)	0.0 (null)	23 (15:15)	11:00 (44.386)	12:03 (15.609)	null (0.0)	11:00 (28.482)	null (0.0)	null (44.386)	0.028	0	124 (0)
**5%**	0.175	0.181	0.028	2.223	2.209	2.56	26.755 (11:00)	7.811 (12:15)	0.0 (null)	23 (15:15)	11:00 (44.406)	12:03 (18.12)	null (0.0)	11:00 (28.507)	null (0.0)	null (44.406)	0.028	0	124 (0)
**10%**	0.187	0.203	0.028	2.262	2.231	2.559	27.457 (11:00)	9.66 (14:00)	0.0 (null)	23 (15:15)	11:00 (45.451)	12:03 (17.86)	13:45 (0.0)	11:00 (28.217)	null (0.0)	null (45.451)	0.028	0	128 (4)
**15%**	0.185	0.201	0.028	2.26	2.229	2.557	27.472 (11:00)	9.518 (14:00)	0.0 (null)	23 (15:15)	11:00 (45.449)	12:03 (17.852)	13:45 (0.0)	11:00 (28.226)	null (0.0)	null (45.449)	0.028	0	127 (3)
**20%**	0.21	0.245	0.029	2.32	2.273	2.561	29.543 (11:00)	12.354 (12:15)	0.0 (null)	25 (14:45)	11:00 (46.277)	12:03 (21.085)	12:15 (0.0)	11:00 (31.066)	null (0.0)	null (46.277)	0.029	1	130 (6)
**25%**	0.221	0.273	0.029	2.356	2.301	2.559	29.784 (11:00)	12.654 (12:15)	0.0 (null)	25 (14:15)	11:00 (43.523)	12:03 (21.427)	12:15 (0.0)	11:00 (31.639)	null (0.0)	null (43.523)	0.03	1	131 (7)
**30%**	0.242	0.33	0.032	2.42	2.359	2.562	32.25 (11:00)	15.652 (12:00)	0.0 (null)	25 (14:30)	11:00 (49.199)	12:05 (21.03)	12:01 (0.0)	11:00 (31.712)	null (0.0)	null (49.199)	0.03	3	135 (11)
**35%**	0.312	0.492	0.034	2.539	2.522	2.565	33.365 (11:00)	18.449 (12:15)	0.0 (null)	28 (14:45)	11:00 (49.472)	12:03 (26.089)	11:00 (0.0)	11:00 (34.341)	null (0.0)	null (49.472)	0.029	5	137 (13)
**40%**	0.354	0.622	0.034	2.613	2.653	2.564	37.235 (11:00)	19.639 (12:15)	0.0 (null)	29 (14:45)	11:00 (50.572)	12:03 (25.51)	11:30 (0.269)	11:00 (34.004)	null (0.0)	14:30 (50.572)	0.032	9	144 (20)
**45%**	0.361	0.646	0.034	2.624	2.676	2.565	36.845 (11:00)	19.818 (12:15)	0.0 (null)	29 (14:45)	11:00 (50.504)	11:00 (26.152)	11:15 (0.0)	11:00 (35.205)	null (0.0)	null (50.504)	0.03	10	143 (19)
**50%**	0.364	0.744	0.065	2.675	2.776	2.595	40.444 (11:00)	22.111 (12:00)	0.0 (null)	28 (15:45)	11:00 (55.496)	11:00 (30.774)	11:30 (0.0)	11:00 (41.512)	null (0.0)	null (55.496)	0.03	18	148 (24)
**Day 2**																			
**0%**	0.514	0.514	null	2.541	2.541	null	46.305 (11:45)	31.866 (12:30)	1.355 (17:15)	31 (17:15)	11:33 (50.352)	12:18 (41.288)	null (0.0)	11:48 (46.744)	15:18 (3.031)	null (50.352)	0.038	0	146 (0)
**5%**	0.597	0.646	0.028	2.664	2.673	2.559	46.668 (11:45)	36.775 (12:30)	2.845 (15:30)	33 (17:30)	11:33 (54.857)	12:18 (44.25)	13:00 (0.0)	11:48 (46.844)	15:18 (6.221)	null (54.857)	0.046	0	153 (7)
**10%**	0.588	0.645	0.03	2.662	2.673	2.56	46.788 (11:45)	36.702 (12:30)	3.846 (15:15)	33 (17:00)	11:33 (51.952)	12:18 (44.251)	12:45 (0.0)	11:48 (46.792)	15:20 (3.713)	null (51.952)	0.048	1	155 (9)
**15%**	0.65	0.747	0.028	2.746	2.776	2.556	47.568 (11:45)	39.45 (12:30)	4.96 (15:30)	34 (17:30)	11:33 (65.171)	12:18 (45.113)	13:00 (0.0)	11:48 (49.022)	15:18 (7.109)	null (65.171)	0.059	0	157 (11)
**20%**	0.845	1.134	0.033	3.006	3.164	2.563	49.997 (11:45)	46.002 (12:15)	11.996 (15:15)	39 (17:30)	11:33 (66.143)	12:18 (46.723)	12:15 (0.0)	11:48 (51.553)	15:03 (17.136)	null (66.143)	0.147	5	168 (22)
**25%**	0.824	1.109	0.033	2.987	3.139	2.563	49.995 (11:45)	46.332 (12:15)	12.182 (15:15)	39 (17:30)	11:33 (66.143)	12:18 (46.903)	12:15 (0.0)	11:48 (50.973)	15:03 (17.266)	null (66.143)	0.144	5	169 (23)
**30%**	0.819	1.204	0.049	3.015	3.234	2.578	50.729 (11:45)	45.112 (12:30)	9.723 (15:15)	38 (17:30)	11:33 (66.914)	12:18 (46.96)	12:30 (0.0)	11:48 (50.767)	15:05 (17.34)	null (66.914)	0.14	7	167 (21)
**35%**	0.878	1.356	0.054	3.092	3.387	2.585	53.225 (11:45)	46.156 (12:30)	15.138 (13:45)	36 (17:30)	11:33 (66.915)	12:20 (50.162)	12:16 (0.0)	11:48 (52.112)	15:18 (16.982)	null (66.915)	0.171	12	174 (28)
**40%**	0.87	1.59	0.047	3.136	3.625	2.577	55.925 (11:30)	46.716 (12:30)	16.544 (15:30)	37 (16:30)	11:33 (66.993)	12:20 (51.765)	12:16 (0.0)	11:48 (56.692)	15:33 (17.146)	null (66.993)	0.18	17	183 (37)
**45%**	0.987	1.83	0.066	3.257	3.863	2.596	56.174 (11:30)	49.787 (12:15)	18.998 (14:45)	38 (16:45)	11:33 (66.988)	12:33 (51.641)	12:16 (4.456)	11:48 (54.967)	15:18 (23.72)	12:46 (66.988)	0.274	22	176 (30)
**50%**	0.961	1.99	0.087	3.264	4.026	2.617	57.365 (11:45)	50.398 (12:30)	20.445 (15:15)	38 (17:30)	11:33 (75.424)	12:18 (53.59)	12:30 (0.0)	11:48 (56.004)	15:03 (25.072)	null (75.424)	0.34	23	186 (40)

PCR—Patient contamination rate, Tot.—Total patient population, Ord.—Ordinary patients without suspicion of being pathogen contagious, Con.—Patients categorized as being with suspicion pathogen contagion, ED—Emergency department, E.Tr.—Extra treatment rooms, Tri.—Triage, WZ—Waiting zone, WR—Waiting room.

## References

[1] Cucinotta D., Vanelli M. (2020). WHO Declares COVID-19 a Pandemic. Acta Biomed..

[2] Ciotti M., Ciccozzi M., Terrinoni A., Jiang W.-C., Wang C.-B., Bernardini S. (2020). The COVID-19 Pandemic. Crit. Rev. Clin. Lab. Sci..

[3] Acuti Martellucci C., Flacco M.E., Cappadona R., Bravi F., Mantovani L., Manzoli L. (2020). SARS-CoV-2 Pandemic: An Overview. Adv. Biol. Regul..

[4] Helse Stavanger S. Koronavirus—Rutiner for Medarbeidere. https://helse-stavanger.no/om-oss/for-ansatte/koronavirus-rutiner-for-ansatte.

[5] Fredheim G. Helse- og Omsorgsdepartementet: Regjeringens Strategi og Beredskapsplan for Håndteringen av COVID-19-Pandemien. https://www.regjeringen.no/no/dokumenter/regjeringens-strategi-og-beredskapsplan-for-handteringen-av-covid-19-pandemien/id2907427/.

[6] Liu Y., Gayle A.A., Wilder-Smith A., Rocklöv J. (2020). The Reproductive Number of COVID-19 Is Higher Compared to SARS Coronavirus. J. Travel Med..

[7] Ribaric N.L., Vincent C., Jonitz G., Hellinger A., Ribaric G. (2022). Hidden Hazards of SARS-CoV-2 Transmission in Hospitals: A Systematic Review. Indoor Air.

[8] People with Certain Medical Conditions. https://www.cdc.gov/coronavirus/2019-ncov/need-extra-precautions/people-with-medical-conditions.html.

[9] Capalbo C., Aceti A., Simmaco M., Bonfini R., Rocco M., Ricci A., Napoli C., Rocco M., Alfonsi V., Teggi A. (2020). The Exponential Phase of the COVID-19 Pandemic in Central Italy: An Integrated Care Pathway. Int. J. Environ. Res. Public Health.

[10] Korber B., Fischer W.M., Gnanakaran S., Yoon H., Theiler J., Abfalterer W., Hengartner N., Giorgi E.E., Bhattacharya T., Foley B. (2020). Tracking Changes in SARS-CoV-2 Spike: Evidence That D614G Increases Infectivity of the COVID-19 Virus. Cell.

[11] Rutherford P.A., Provost L.P., Kotagal U.R., Luther K., Anderson A. (2017). Institute for Healthcare Improvement: Achieving Hospital-Wide Patient Flow. https://www.ihi.org/resources/Pages/IHIWhitePapers/Achieving-Hospital-wide-Patient-Flow.aspx.

[12] McHugh M., VanDyke K., McClelland M., Moss D. (2012). Improving Patient Flow and Reducing Emergency Department Crowding: A Guide for Hospitals.

[13] Mason S. (2011). Keynote Address: United Kingdom Experiences of Evaluating Performance and Quality in Emergency Medicine. Acad. Emerg. Med..

[14] Mohiuddin S., Busby J., Savović J., Richards A., Northstone K., Hollingworth W., Donovan J.L., Vasilakis C. (2017). Patient Flow within UK Emergency Departments: A Systematic Review of the Use of Computer Simulation Modelling Methods. BMJ Open.

[15] Vanbrabant L., Braekers K., Ramaekers K., Van Nieuwenhuyse I. (2019). Simulation of Emergency Department Operations: A Comprehensive Review of KPIs and Operational Improvements. Comput. Ind. Eng..

[16] Bansal K., Kumar S. (2022). Mutational Cascade of SARS-CoV-2 Leading to Evolution and Emergence of Omicron Variant. Virus Res..

[17] Bhattacharjee P., Ray P.K. (2014). Patient Flow Modelling and Performance Analysis of Healthcare Delivery Processes in Hospitals: A Review and Reflections. Comput. Ind. Eng..

[18] Nataraja R.M., Oo Y.M., Kyaw K.K., Webb N.R., Ljuhar D., Pacilli M., Win N.N., Kimber C., Aye A. (2020). Clinical Impact of the Introduction of Pediatric Intussusception Air Enema Reduction Technology in a Low- to Middle-Income Country Using Low-Cost Simulation-Based Medical Education. Simul. Healthc..

[19] Aljahany M., Alassaf W., Alibrahim A.A., Kentab O., Alotaibi A., Alresseeni A., Algarni A., Algaeed H.A., Aljaber M.I., Alruwaili B. (2021). Use of In Situ Simulation to Improve Emergency Department Readiness for the COVID-19 Pandemic. Prehosp. Disaster Med..

[20] Salmon A., Rachuba S., Briscoe S., Pitt M. (2018). A Structured Literature Review of Simulation Modelling Applied to Emergency Departments: Current Patterns and Emerging Trends. Oper. Res. Health Care.

[21] Castanheira-Pinto A., Gonçalves B.S., Lima R.M., Dinis-Carvalho J. (2021). Modeling, Assessment and Design of an Emergency Department of a Public Hospital through Discrete-Event Simulation. Appl. Sci..

[22] Hamza N., Majid M.A., Hujainah F. (2021). SIM-PFED: A Simulation-Based Decision Making Model of Patient Flow for Improving Patient Throughput Time in Emergency Department. IEEE Access.

[23] Hamza N., Abdul Majid M., Adam K., Akma Abu Bakar N. (2019). A Review on Simulation and Modelling for Patient Flow in Emergency Department. IOP Conf. Ser. Mater. Sci. Eng..

[24] Friesen M.R., McLeod R.D. (2014). A Survey of Agent-Based Modeling of Hospital Environments. IEEE Access.

[25] Terning G., Brun E. Systemic Conceptual Modeling of Patient Flow in a Hospital Emergency Department: A Case Example. Proceedings of the System Dynamics Society Record of the 38th International Conference of the System Dynamics Society.

[26] Randers J. (1997). Elements of the System Dynamics Method. J. Oper. Res. Soc..

[27] Albin S., Forrester J.W., Breierova L. (2001). Building a System Dynamics Model: Part 1: Conceptualization.

[28] Luna L.F., Andersen D.L. Using Qualitative Methods in the Conceptualization and Assessment of System Dynamics Models. Proceedings of the 20th International System Dynamics Conference.

[29] Terning G., Brun E.C., El-Thalji I. (2023). The Patient Flow Effect of Pandemic Policies: A Hybrid Simulation Study in a Norwegian Emergency Department. Healthcare.

[30] Borshchev A. (2013). The Big Book of Simulation Modeling: Multimethod Modeling with AnyLogic 6.

[31] Ören T., Zeigler B.P., Tolk A. (2023). Body of Knowledge for Modeling and Simulation: A Handbook by the Society for Modeling and Simulation International.

[32] Terning G., Brun E.C., El-Thalji I. (2022). Modeling Patient Flow in an Emergency Department under COVID-19 Pandemic Conditions: A Hybrid Modeling Approach. Healthcare.

[33] Suh H. Om oss—Stavanger Universitetssjukehus—Helse Stavanger HF. https://helse-stavanger.no/om-oss.

[34] Minge A. Hun Skal Hele Tiden Være Orakelet og ta Raske og Rette Avgjørelse. Denne Dagen Varte Pausen i 30 Sekunder. https://www.aftenbladet.no/magasin/i/EWgGxa/hun-skal-hele-tiden-vaere-orakelet-og-ta-raske-og-rette-avgjoerelse-den.

[35] Cho Y.-J., Yeo I.-H., Lee D.-E., Kim J.-K., Kim Y.-J., Kim C.-H., Choe J.-Y., Park J.-B., Seo K.-S., Yu B.-H. (2022). Collateral Effect of the Coronavirus Disease 2019 Pandemic on Emergency Department Visits in Korea. Medicina.

[36] Cummins N.M., Garavan C., Barry L.A., Devlin C., Corey G., Cummins F., Ryan D., McCarthy G., Galvin R. (2022). The Impact of COVID-19 on an Irish Emergency Department (ED): A Cross-Sectional Study Exploring the Factors Influencing ED Utilisation Prior to and during the Pandemic from the Patient Perspective. BMC Emerg. Med..

[37] Krogstad U., Lindahl A., Saastad E., Hafstad E. (2015). Akuttmottak-En Risikosone for Pasientsikkerhet. Læringsnotat Fra Meldeordningen i Kunnskapssenteret 2015.

[38] Skinner J., Higbea R., Buer D., Horvath C. (2018). Using predictive analytics to align ED staffing resources with patient demand: A hospital in Grand Rapids, Mich., used management theory and data analysis to design and implement a much more precise model for setting staffing levels in its emergency department. Healthc. Financ. Manag..

[39] Phan T., Brozak S., Pell B., Gitter A., Xiao A., Mena K.D., Kuang Y., Wu F. (2023). A Simple SEIR-V Model to Estimate COVID-19 Prevalence and Predict SARS-CoV-2 Transmission Using Wastewater-Based Surveillance Data. Sci. Total Environ..

